# Progesterone receptor membrane component 1 is involved in oral cancer cell metastasis

**DOI:** 10.1111/jcmm.15535

**Published:** 2020-07-16

**Authors:** Hsun‐Yu Huang, Hsiu‐Chuan Chou, Ching‐Hsuan Law, Wan‐Ting Chang, Tzu‐Ning Wen, En‐Chi Liao, Meng‐Wei Lin, Li‐Hsun Lin, Yu‐Shan Wei, Yi‐Ting Tsai, Hsin‐Yi Chen, Kui‐Thong Tan, Wen‐Hung Kuo, Mei‐Lan Ko, Shing‐Jyh Chang, Ying‐Ray Lee, Hong‐Lin Chan

**Affiliations:** ^1^ Dental Department of Dimanson Medical Foundation Chia‐Yi Christian Hospital Chia‐Yi Taiwan; ^2^ Institute of Analytical and Environmental Sciences National Tsing Hua University Hsinchu Taiwan; ^3^ Department of Medical Science and Institute of Bioinformatics and Structural Biology National Tsing Hua University Hsinchu Taiwan; ^4^ Department of Chemistry National Tsing Hua University Hsinchu Taiwan; ^5^ Department of Surgery National Taiwan University Hospital Taipei Taiwan; ^6^ Department of Biomedical Engineering and Environmental Sciences National Tsing Hua University Hsinchu Taiwan; ^7^ Department of Ophthalmology National Taiwan University Hospital Hsin‐Chu Branch Hsinchu Taiwan; ^8^ Department of Obstetrics and Gynecology Hsinchu MacKay Memorial Hospital Hsinchu Taiwan; ^9^ Department of Medical Research Ditmanson Medical Foundation Chia‐Yi Christian Hospital Chiayi Taiwan

**Keywords:** metastasis, oral cancer, progesterone receptor membrane component 1, proteomics

## Abstract

Cancer metastasis is a common cause of failure in cancer therapy. However, over 60% of oral cancer patients present with advanced stage disease, and the five‐year survival rates of these patients decrease from 72.6% to 20% as the stage becomes more advanced. In order to manage oral cancer, identification of metastasis biomarker and mechanism is critical. In this study, we use a pair of oral squamous cell carcinoma lines, OC3, and invasive OC3‐I5 as a model system to examine invasive mechanism and to identify potential therapeutic targets. We used two‐dimensional differential gel electrophoresis (2D‐DIGE) and matrix‐assisted laser desorption ionization time‐of‐flight mass spectrometry (MALDI‐TOF/TOF MS) to examine the global protein expression changes between OC3 and invasive OC3‐I5. A proteomic study reveals that invasive properties alter the expression of 101 proteins in OC3‐I5 cells comparing to OC3 cells. Further studies have used RNA interference technique to monitor the influence of progesterone receptor membrane component 1 (PGRMC1) protein in invasion and evaluate their potency in regulating invasion and the mechanism it involved. The results demonstrated that expression of epithelial‐mesenchymal transition (EMT) markers including Twist, p‐Src, Snail1, SIP1, JAM‐A, vimentin and vinculin was increased in OC3‐I5 compared to OC3 cells, whereas E‐cadherin expression was decreased in the OC3‐I5 cells. Moreover, in mouse model, PGRMC1 is shown to affect not only migration and invasion but also metastasis in vivo. Taken together, the proteomic approach allows us to identify numerous proteins, including PGRMC1, involved in invasion mechanism. Our results provide useful diagnostic markers and therapeutic candidates for the treatment of oral cancer invasion.

## 1 INTRODUCTION

Oral cancer occurring often in oral cavity belongs to head and neck cancer. Tumours at these sites classically cause drinking and eating difficulties. Alcohol, tobacco and betel quid consumption are well‐reported risk factors for more than 90% oral cancer cases.^1^ More than nine in ten of cancer deaths are attributable to cancer metastatic disease that has moved to the centre of clinic attention. Metastasis is a multistage process in which cancer cells spread out from primary site to another non‐adjacent part. This spread of cancer is formed by a complex and dynamic series of cell‐biological events.^2^ These invasive cancer cells invade locally through the extracellular matrix and the lymphatic or blood vessels, and they enter the circulation system. Subsequently, the circulating tumour cells survive in the absence of anchorage and are transported in the circulation system. Cancer cells lodge in the microvasculature of a non‐adjacent site, cross from the vessels into the tissue, and survive and proliferate to form metastatic colonies in a foreign microenvironment, eventually; hence, metastatic cancer cells in each step are phenotypically and functionally distinct from primary tumour cells and cells in other steps. For the purpose of improving patient survival rate, metastasis prevention has become one of the most important clinical issues. To comprehend the metastasis mechanism and to find out the main metastasis‐regulating factors are the best way to solve this issue.

There are over 60% of oral cancer patients present with advanced stage disease, and the five‐year survival rates of these patients decrease from 72.6% to 20% as the stage becomes more advanced.^1^ The advanced diseases commonly involve regional lymph nodes metastasis. The distant metastasis of oral cancer cells is rare, arising in 17% of patients, and mostly metastasized to lung following by the mediastinal lymph nodes, liver and bones. Despite the rapid progression in diagnosis and treatment of head and neck cancer during last two decades, only a slight improvement in the 5‐year survival rate for oral cancer patients.^3^ And at least 50% of patients with regional and distant diseases develop locoregional or distant relapses in the first 2 years after treatment. The disappointed results are considered to be relevant to the highly invasive and metastatic properties of oral cancer.

These clinical realities have been known for decades. But the invasion and metastasis cell‐biolocal progression of oral cancer has emerged only recently, and remains largely unknown. To effectively manage oral cancer metastasis, the cell‐biological and molecular changes established in oral cancer model are needed. In which, the identification of metastasis biomarker and mechanism is critical. At present, high‐throughput experiments are widely used in the discovery of cancer metastatic biomarker. Previously, in transcriptomic field, several studies have set up the gene expression profiles in oral cancer metastasis by cDNA microarray.^4^, ^5^, ^6^ There are also several proteomic studies which reveal the protein expression level of metastasis and non‐metastasis oral cancer, and most of those studies used oral cancer patients' tumour tissues as materials. Several metastasis‐related protein candidates have been found, such as superoxide dismutase 2 (SOD2)^7^ and caldesmon.^8^ However, clinical samples exhibit different gene backgrounds from patient to patient and lack of homogeneity, which may shadow the potential metastasis‐related proteins and biomarkers. In this study, we used OSCC cell lines OC3 and OC3‐I5 with different metastatic potential derived from a single patient as materials to limit the heterogeneity. We analysed the cytosolic and secreted protein abundance of both invasive and non‐invasive OSCC by using 2D‐DIGE, and identified the proteins with different expression levels by MALDI‐TOF MS. A total of 101 differently expressed proteins were identified. Among all the metastasis‐related candidates, membrane‐associated progesterone receptor component 1 (PGRMC1) was selected for further investigation and show potency as an oral cancer therapeutic targets. By using oral cancer cells carrying a shRNA to inactivate the PGRMC1 gene function, we addressed the role of PGRMC1 in the development of tumour metastasis to the lung in vivo. We demonstrated that the development and metastasis of the oral cancer OC3 cells in vivo depend substantially on PGRMC1 expression.

## 2 MATERIALS AND METHODS

### 2.1 Chemicals and reagents

Lipofectamine RNAiMAX reagent and OPTI‐MEM were purchased from Life Technologies; 3‐(4,5‐dimethylthiazol‐2‐yl)‐2,5‐diphenyl tetrazolium bromide (MTT) was purchased from USB Corporation; CellTiter Blue was purchased from Promega Corporation; Primary antibodies were purchased from Genetex; antimouse and anti‐rabbit secondary antibodies were purchased from GE Healthcare; fluorescein isothiocyanate–conjugated anti‐rabbit immunoglobulin G antibody was purchased from Jackson Immuno Research Laboratories; foetal bovine serum (FBS), trypsin‐EDTA, Dulbecco modified Eagle medium (DMEM), and penicillin and streptomycin (P/S) were purchased from Gibco‐Invitrogen Corporation; 10‐, 15‐ and 4‐cm Petri dishes and 96‐ and 24‐well plates were purchased from Orange Scientific; MTT, Tris base, glycerol, NP‐40, sodium dodecyl sulphate (SDS) and tetramethylethylenediamine were purchased from USB Corporation; bovine serum albumin was purchased from Sigma‐Aldrich; Horseradish peroxidase‐linked anti‐rabbit IgG was purchased from GE Healthcare; Tris HCl (pH 6.8) was purchased from Severn Biotech Ltd; HCl was purchased from Scharlau Chemie; repel solution and cover oil were purchased from GE Healthcare; and an enhanced chemiluminescence substrate kit was purchased from Visual Protein Corporation. All chemicals and biochemicals used in this study were of an analytical grade.

### 2.2 Cell lines and cell cultures

OC3 and OC3‐I5 oral cancer cells were received as a gift from Dr Wang Lu‐Hai. The OC3‐I5 cells, which were OC3 cells selected from a transwell invasion assay, were reselected every 10 passages to maintain their invasive ability. The detail culture information of OC3 and OC3‐I5 cells was reported in our previous publication.^9^

### 2.3 MTT cell viability assay

The OC3 and OC3‐I5 cells were seeded into 96‐well plates at a density of 7000 cells/well for performing MTT assay. The detail procedure for the assay has been described in our previous publication.^9^

### 2.4 Immunoblotting assay

Immunoblotting was employed to validate the differential expression of mass spectrometry‐identified proteins. The detail procedure for the assay has been described in our previous publication.^9^

### 2.5 Enzyme‐linked immunosorbent assay (ELISA)

EIA polystyrene microtiter plates were coated with 50 μg of protein lysate sample and incubated at 37°C for 2 hours. The plate was washed three times with phosphate buffered saline with Tween‐20 (PBS‐T) and three times with PBS. Plates were then blocked with 100 μL of 5% skimmed milk in PBS at 37°C for 2 hours and then washed three times with PBST. Antibody solution was added and incubated at 37°C for 2 hours. After washing with PBST and PBS for 10 times in total, 100 μL of peroxidise‐conjugated secondary antibody in PBS was added for incubation at 37°C for 2 hours. Following 10 washes, 100 μL of 3,3,5,5‐tetramethyl benzidine (Pierce) was added. After incubation at room temperature for 30 minutes, 100 μL of 1 M H_2_SO_4_ was added to stop the reaction and the absorbance at 450 nm measured using a Stat Fax 2100 microtiter plate reader (Awareness Technology Inc).

### 2.6 siRNA design, shRNA cell line establishment and transfection

The targeting sequences 5′‐AAU UUG CGG CCU UUG GUC ACA UCG A‐3′ and 5′‐AGU GAA CUG AGA CUC CCA GUC ACU C‐3′ against PGRMC1 were designed and synthesized by Invitrogen. The sequences with similar GC content were used as the negative control against PGRMC‐1 (Invitrogen). The OC3 and OC3‐I5 cells were transfected with 60 nMPGRMC1 small interfering RNA (siRNA) or the corresponding scramble control (pGCsi) by using the Lipofectamine RNAiMAX transfection reagent (Invitrogen) according to the manufacturer's instructions. All PGRMC‐1 cell lines were grown to 50% confluence before transfection. 10nM of plasmid DNA was transfected in OC3 and OC3‐I5 following OC3 and OC3‐I5 cells were transfected with 10nM of shPGRMC1 plasmid DNA or shLacZ plasmid control by Lipofectamine^®^ LTX (Invitrogen). OC3 and OC3‐I5 cells were at 50% confluence at the time of transfection. Lipofectamine^®^ LTX was diluted in OPTI‐MEM^®^, and shRNA was diluted in OPTI‐MEM^®^ separately. After 5 minutes mixed the diluted Lipofectamine^®^ LTX into diluted shRNA in a 1:1 ratio and incubate for 20 minutes. Added shRNA‐Lipofectamine^®^ to cells in serum‐free medium. Incubated the cells 4 hours at 37°C in incubator and changed the serum‐free medium to complete medium. Recovered for 24 hours and repeated the transfect steps again.

Stable cell lines could be established by puromycin selection after transfection. shPGRMC1 or shLacZtransfectedOC3 and OC3‐I5 cells were selected by their resistance to puromycin. One day after subcultured, puromycin was added in transfected OC3 and OC3‐I5 cells at a concentration of 1 μg/mL. Cells were incubated with puromycin‐containing medium for 48 hours and recovered in serum medium for 48 hours. The puromycin selections were performed every two passages until the cell viability would not affected by puromycin.

### 2.7 Flow cytometry analysis for apoptosis detection and cell‐cycle analysis

For apoptosis detection, the percentage of apoptotic cells stained using the FITC Annexin V Apoptosis Detection kit I (BD Biosciences) was determined. The detail procedure for the assay has been described in our previous publication.^9^ Data were further analysed using Cflow Plus analysis software (BD Biosciences).

### 2.8 Migration and invasion assay

Transwell assay was employed to examine the effect of PGRMC1 on migration and invasion in OC3/ OC3‐I5 cells. Transwell cell culture inserts (PET membrane) with a pore size of 8.0 µm (SPL Life Sciences) were coated or not coated with Matrigel™ (BD Biosciences) in an FBS‐free DMEM medium. The detail procedure for the assay has been described in our previous publication.^9^ The cells that had invaded were visible under the optical microscope and were observed at a magnification of 100X.

### 2.9 Immunofluorescence staining

For immunofluorescence staining of OC3 and OC3‐I5 cells, the detail procedure for the assay has been described in our previous publication.^9^ Images were exported in the TIF file format by using Zeiss Axiovision version 4.8 and processed using Adobe Photoshop version 7.0 (Adobe Systems).

### 2.10 Wound‐healing migration assay

Wound‐healing assay provides an easy and simple means for monitoring directional cell migration and interaction in vitro. The detail procedure for the assay has been described in our previous publication.^9^ The wound areas were calculated using AxioVision version 4.

### 2.11 MTT cell viability assay

The detail procedure for the assay has been described in our previous publication.^9^

### 2.12 Orthotopic tumour implantation

Five‐week‐old BALB/cAnN.Cg‐Foxnlnu/CrlNarl female mice were housed in a specific‐pathogen‐free environment. One week after housing, mice were injected with human oral cancer cells. 3 × 10^6^ OC3 and OC3‐I5 cells stably transfected with shPGRMC1 or shLacZ were mixed with Matrigel™ in PBS (1:7) and inoculated subcutaneously onto the right tight of mice (6 per group). The mice were killed by zoletil/rompun60 days after implantation, and the lungs were removed and fixed in 10% formalin. For further analysis, the lung samples were tissue trimming, tissue processing and embedding, section and H & E staining to examine tissue morphologies, which were performed by National Laboratory Animal Center, Taiwan, ROC All mice were fed ad lib and housed according to the guidelines of the Society of Laboratory Animal Science.

### 2.13 Statistical analysis

The Student *t* test and analysis of variance were employed for the statistical analysis, with *P* < .05 considered statistically significant.

## 3 RESULTS

### 3.1 Invasion properties of OC3‐I5 cells compared to OC3 cells

**FIGURE 1:**
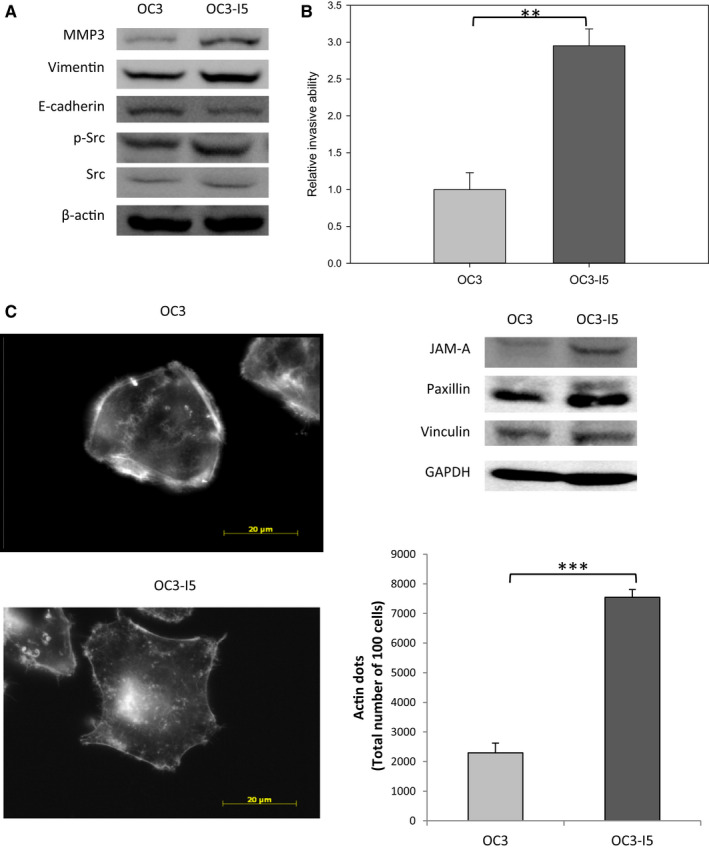
The invasive properties and focal adhesion formation of OC3‐I5 cells compared to OC3 cells. (A) The expression levels of EMT markers vimentin, E‐cadherin and matrix metalloproteinase‐3 (MMP3) in OC3 and OC3‐I5 cells were presented by immunoblotting. β‐actin was used as loading control. (B) The invasion ability of OC3 and OC3‐I5 cells was monitored by transwell invasion assay. 10^5^ cells in the matrix‐coated upper chamber of the transwell inserts, and incubated at 37°C for 18 hours. After incubation, cell was fixed in 4% paraformaldehyde and stained with crystal violet. The crystal violet was dissolved in ethanol/acetic acid solution, and the OD value at 600 nm of the solution was measured. OC3‐I5 cells showed 2.7‐fold better invasion ability than OC3 cells. Error bars denote mean ± SEM (n = 3). **P < .01. (C) Cells were seeded on 12 mm glass slides. After fixed in 4% paraformaldehyde and permeabilized with 0.5% Triton X‐100, cells were hybridized with phalloidin. Images were photographed at the magnification of 4000× by microscopy. The actin dots are indicated by yellow arrows. Scale bar, 20 μm. The expression levels of migration markers were monitored by immunoblotting. GAPDH was used as loading control. The actin dots in OC3 and OC3‐I5 cells were counted and the quantified. OC3‐I5 cells formed more actin dots than OC3 cells at about 3.3‐fold. Error bars denote mean ± SEM (n = 3). ***P < .001

To evaluate the invasion mechanism of oral cancer, we prepared a human oral cancer cell line, OC3, and an invasive oral cancer cell line, OC3‐I5. OC3‐I5 cells were derived fromOC3 cells and selected through transwell invasion assay. After every ten passages, OC3‐I5 cells were re‐transwell selected to maintain the invasive ability. Transwell invasion assay is a widely used platform for measuring invasion ability. The transwell inserts create a two‐chamber system separated by one layer of cell‐permeable plastic membrane. Matrigel™ coating on the cell‐permeable membrane imitates the extracellular matrix that cells would encounter during invasion in vivo. The invasive cells would enzymatically degrade Matrigel™ barrier and migrate through the pore on membrane towards chemoattractant in lower chamber. The invasion ability was measured by counting those cells which had traversed cell‐permeable membrane. By transwell invasion assay, OC3‐I5 cells showed better invasion ability than OC3 cells at about 2.7‐fold (Figure 1B).

In the course of investigating whether OC3‐to‐OC3‐I5 transition is a result of epithelial‐mesenchymal transition, we examined the epithelial‐mesenchymal transition markers E‐cadherin, vimentin and matrix metalloproteinase‐3 (MMP3) in OC3 and OC3‐I5 cells via immunoblotting. OC3‐I5 cells expressed lower level of epithelial marker E‐cadherin, but higher level of mesenchymal marker vimentin, and MMP3 than OC3 cells (Figure 1A). This observation indicated that OC3‐I5 cells were at a more mesenchymal cell status than OC3 cells, which confirmed the previous results of invasion assay.

In cells that exhibit movement, a significant part of the migration cycle is the formation of stable attachment points called focal adhesions. Focal adhesions can transduce forces between cytoskeleton and the extracellular matrix (ECM) and execute directional movement via the polarization signalling. OC3‐I5 cells presented smaller but much more focal adhesions (actin dots) than OC3 cells did (Figure 1C) and expressed higher level of focal adhesion proteins JAM‐A, paxillin and vinculin (Figure 1C). These data implied that OC3‐I5 cells have better ability of migration than OC3 cells. Moreover, the high level of pSrc (Figure 1A) in OC3‐I5 cells corresponded with focal adhesion data.

### 3.2 Identification and functional characterization of cytosolic proteins involved in human oral cancer invasion

To investigate more detailed mechanisms of oral cancer metastasis, we used 2D‐DIGE and MALDI‐TOF MS/MS to identify the proteins which were involved in oral cancer invasion. OC3 and OC3‐I5 cells were lysed, and proteins were labelled by CyDyes and separated based on the isoelectric point and the molecule weight. Each condition was visualized via Ettan DIGE Imager. The representative 2D‐DIGE images of OC3 and OC3‐I5 cytosolic protein are shown in Figure S1A. After analysing 2D‐DIGE images by DeCyder software version 7.0 and filtering out the dust or background on the basis of spot slope, volume and area, we discovered 1789 well‐defined protein spots. The Student *t* test value ≤ 0.05 was considered and the spots with the mean value ± 1.3‐fold increase or decrease were chosen. 153 spots were chosen as interest, and 133 spots were picked for further identification. The picked spots of interest were digested by trypsin which cleaves protein chain at the carboxyl side of arginine and lysine residues. The fragmented proteins (peptides) were analysed and identified via peptidemass fingerprint (PMF) by MALDI‐TOF MS. 104 differentially expressed protein spots had been characterized (Figure S1B; Table S1) representing as 91 individual proteins. The identified proteins were categorized according to KEGG and Swiss‐Prot database. Most of proteins are cytosolic protein (up to 60%) and are involved in cytoskeleton (17%), protein degradation (7%), protein folding (7%), glycolysis (6%), redox regulation (6%), vesicle trafficking (6%) and so on (data not shown).

### 3.3 Validation of characterized invasion associated proteins via immunoblotting and ELISA analysis

To further validate the expression trend of identified protein, we performed immunoblotting and ELISA analysis of the differentially expressed proteins between OC3 and OC3‐I5 cells. Contrast to OC3 cells, OC3‐I5 cells up‐regulated proteins such as galectin‐1, alpha‐enolase (Enolase‐1), guanine deaminase (Guanase), collagenase 3 (MMP13), calcium‐binding mitochondrial carrier protein SCaMC‐1 (SCaMC‐1), cAMP‐dependent protein kinase catalytic subunit PRKX (PRKX), nuclear distribution protein nudE homolog 1 (NDE1), anamorsin (CIAPIN1), cytochrome P450 2J2 (CYP2J2), glial fibrillary acidic protein (GFAP), superoxide dismutase [Mn] mitochondrial (MnSOD), membrane‐associated progesterone receptor component 1 (PGRMC1), cathepsin D and plastin‐2. Moreover, annexin A2, annexin A3, heat shock 70 kDa protein 1A/1B (Hsp70 1A/1B) and CD63 antigen (CD63) were shown down‐regulated in OC3‐I5 cells (Figure S2). These immunoblotting and ELISA analysis approved the 2D‐DIGE results.

### 3.4 PGRMC1 is required for human oral cancer invasion and migration by regulating EMT via SIP1, Snai1 and Twist transcription factors

Among all the metastasis‐related candidates, membrane‐associated progesterone receptor component 1 (PGRMC1) was selected for further investigation. To investigate the metastatic roles of PGRMC1, PGRMC1 knockdown experiments were performed and two strains of siRNA against PGRMC1 were synthesized by Invitrogen. The sequences 5′‐AAU UUG CGG CCU UUG GUC ACA UCG A‐3′ and 5′‐AGU GAA CUG AGA CUC CCA GUC ACU C‐3′ were designed against PGRMC1. Knockdown of PGRMC1 with the 25 nM of siPGRMC1 showed greater than 90% efficiency in reduction of PGRMC1 protein level, and 50 nM of siPGRMC1 was determined to be used in further investigation (Figure S3).

**FIGURE 2:**
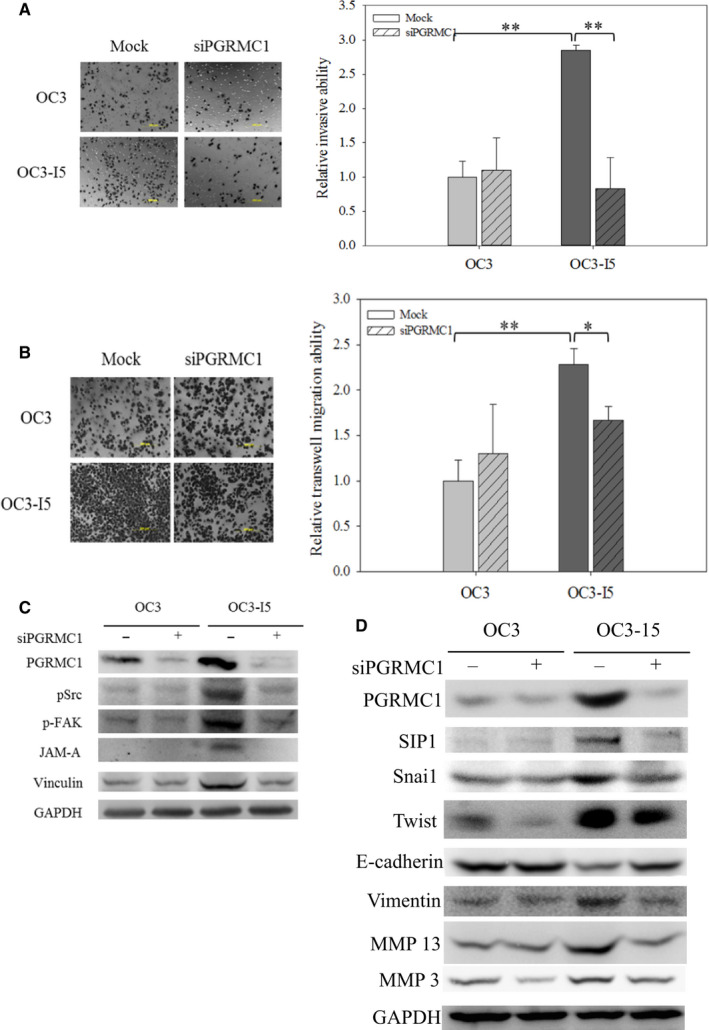
Effects of PGRMC1 knockdown on cell migration and cell invasion in oral cancer cells. (A) OC3 and OC3‐I5 cells were transfected with 50 nM siPGRMC1 or scramble siRNA (mock). 10^5^ cells in serum‐free media were seeded into upper chamber with Matrigel coated. After incubation for 18 hours, cells were fixed and stained by crystal violet and viewed microscopically. The crystal violet were dissolved in ethanol/acetic acid solution and quantified. Mock was regarded as negative control. Error bars denote mean ± SEM (n = 4). **P < .01. (B) Cells were transfected with siPGRMC1 and recovered for 24 hours. Cells were seeded into upper chamber without Matrigel in serum‐free media. After 18 hours, the migratory cells were fixed, stained by crystal violet and viewed microscopically. The crystal violet were dissolved in ethanol/acetic acid solution and quantified. Mock was regarded as negative control. Error bars denote mean ± SEM (n = 3). *P < .05, **P < .01. (C) Total cell lysates were prepared from untreated or siCYP2J2‐treated cells. The phosphorylation levels of Src (tyrosine residue 416) and FAK (tyrosine residue 576/577) were investigated. The downstream proteins, JAM‐A and Vinculin, were also examined. GAPDH was used as loading control. (D) Cell lysates were immunoblotted with antibodies against EMT transcription factors, SIP1, Snai1 and Twist, and downstream proteins, E‐cadherin, Vimentin and MMPs. GAPDH was regarded as loading control

PGRMC1 is a haem‐binding protein with Src homology 2 domain (SH2) and Src homology 3 domain (SH3) binding sites. PGRMC1 is a small protein with a molecular weight of 28 kDa. In normal tissues, PGRMC1 increases lipid synthesis by binding and activating P450 proteins,^10^ while in tumour cells, PGRMC1 deeply affects cell signalling.^11^ PGRMC1 protein has been reported to be overexpressed in several cancer cell lines and tissues, such as breast, thyroid, colon, ovary and lung.^12^ This protein is considered to play a role in tumour promotion and chemotherapy resistance by regulating antiapoptotic pathway.^13^ However, little is known about the relationship between PGRMC1 and cancer invasion, and how PGRMC1 functions in invasion. To examine the role of PGRMC1 in oral cancer invasion, we used siRNA to down‐regulate the expression of PGRMC1. In Figure 2A, the invasion assay revealed that the interference with PGRMC1 inhibited invasion in OC3‐I5 cells compared to OC3‐I5 cells with scramble siRNA transfected control (mock).

Two migration assays in the present of a chemotactic gradient (transwell migration assay) or absence of a chemotactic gradient (wound‐healing migration assay) were also performed in siPGRMC1 knockdown cells. In transwell migration assay, we found that knockdown of PGRMC1 reduced the migration ability of OC3‐I5 cells under chemotactic gradient (Figure 2B).

Migration‐associated phosphorylation on Src and FAK protein and the expression of focal adhesion proteins, JAM‐A and vinculin, in OC3 and OC3‐I5 cells transfected with or without siPGRMC1 were validated by immunoblotting. The phosphorylation levels of Src and FAK and the protein expression levels of JAM‐A and vinculin in OC3‐I5 cells transfected with siPGRMC1 decreased obviously, which highly correlated with migration assays (Figure 2C).

Subsequently, we examined the EMT status in PGRMC1 knockdown cells. The expression levels of EMT‐inducing transcription factors SIP1, Snai1 and Twist were shown in Figure 2D. The interference with PGRMC1 in OC3‐I5 cells reduced the expression of SIP1, Snai1 and Twist. These EMT‐inducing transcription factors would down‐regulate the expression of E‐cadherin, and up‐regulate vimentin and MMPs. The conformable results of E‐cadherin, vimentin and MMPs expression with or without the transfection of siPGRMC1 were also shown in Figure 2D. Altogether, these results demonstrated that PGRMC1 was required for human oral cancer invasion and migration via regulating EMT‐inducing transcription factors SIP1, Snai1 and Twist.

**FIGURE 3:**
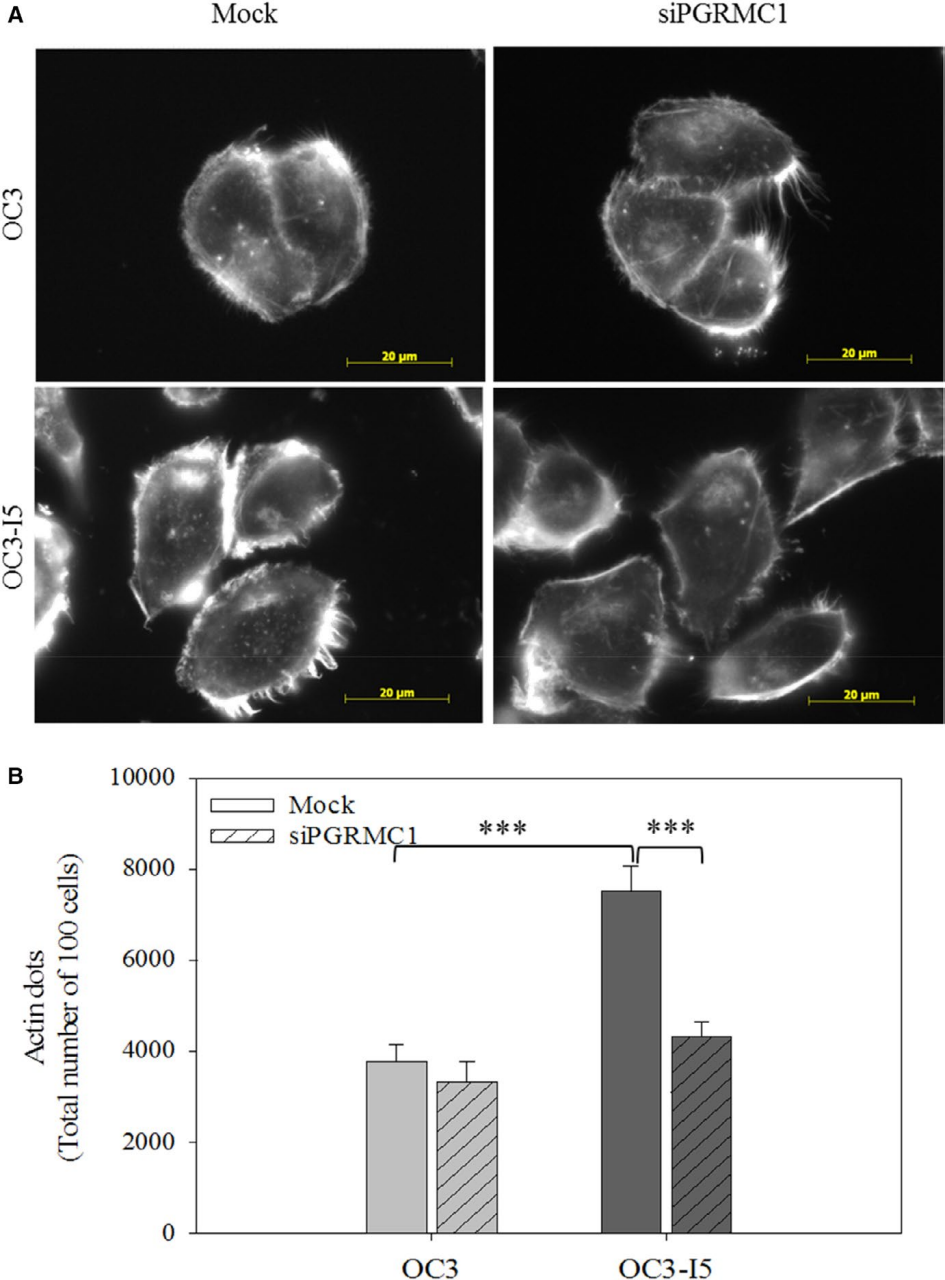
The defect of focal adhesion formation in siPGRMC1 treated oral cancer cells. Cells were transfected with siPGRMC1 and recovered for 24 hours. Cells were seeded on the 12‐mm glass slide and incubated overnight. Cells were fixed and hybridized with phalloidin for 1 hour. (A) The actin dots were photographed and counted microscopically. The actin dots are indicated by yellow arrows. (B) The quantitative data were shown. Mock was regarded as negative control. Scale bar, 20 μm. Error bars denote mean ± SEM (n = 3). ***P < .001

**FIGURE 4:**
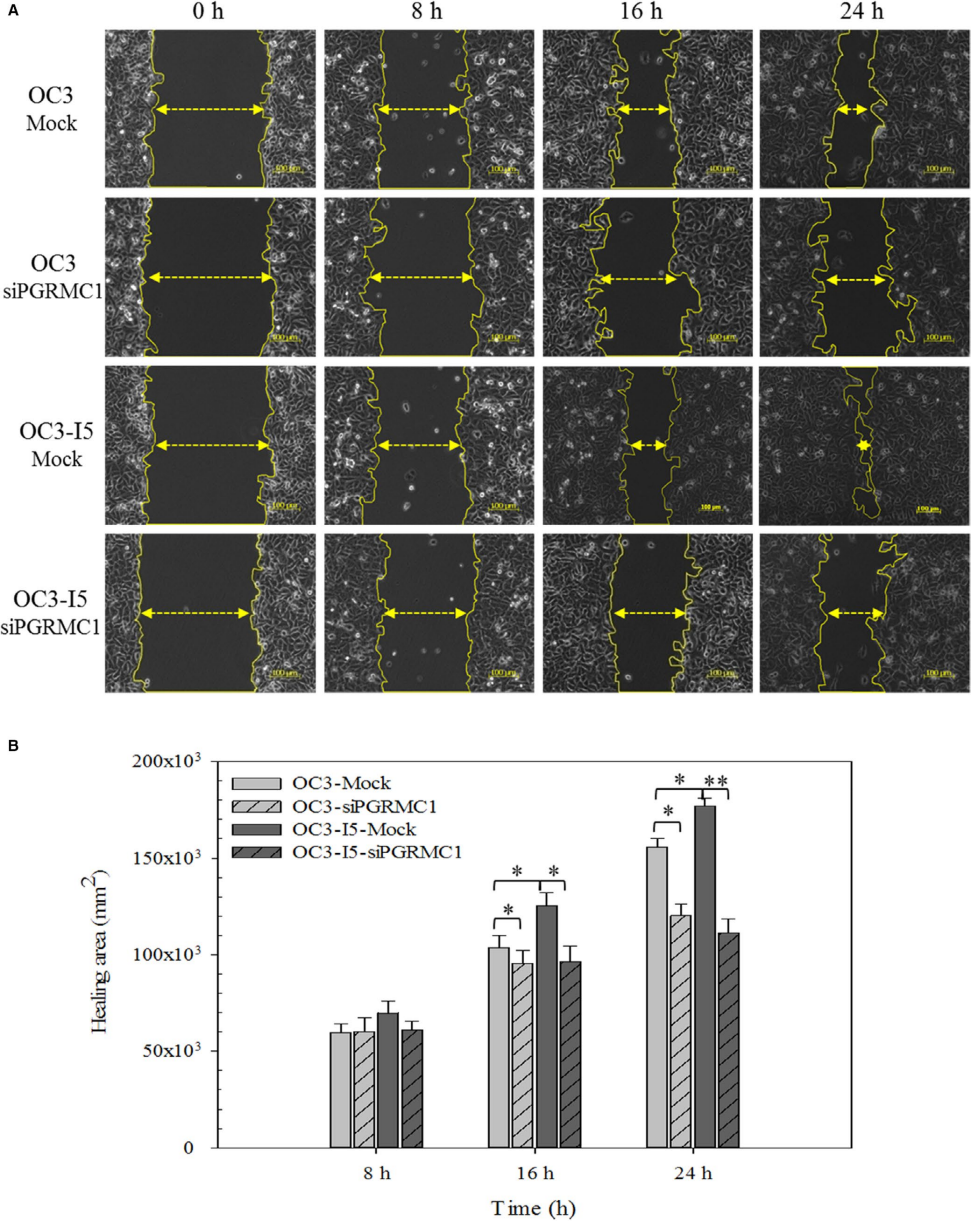
Effect of PGRMC1 knockdown on wound‐healing migration in oral cancer cells. Cells were treated with siRNA and were seeded in 12‐well dish. (A) After overnight incubation, the wounds were created and the wounded cells were photographed at the indicated times. The yellow arrows indicate the wound distance. (B) The relative wound‐healing rates between 8 hours/0 hour, 16 hours/0 hour and 24 hours/0 hour are calculated by Axio Vision 4. Scale bar, 100 μm. Error bars denote mean ± SEM (n = 3). *P < .05, **P < .01

OC3 and OC3‐I5 cells transfected with siPGRMC1 showed a great decrease in migratory ability in the absence of a chemotactic gradient. As expected, siPGRMC1 knockdown OC3‐I5 cells presented less actin dots than mock cells (Figure 3). Additionally, we found that knockdown of PGRMC1 reduced the wound‐healing ability of OC3‐I5 cells in wound‐healing assay (Figure 4).

### 3.5 PGRMC1 is essential for oral cancer proliferation by promoting entry of cells into G2/M phase via p53 down‐regulation

**FIGURE 5:**
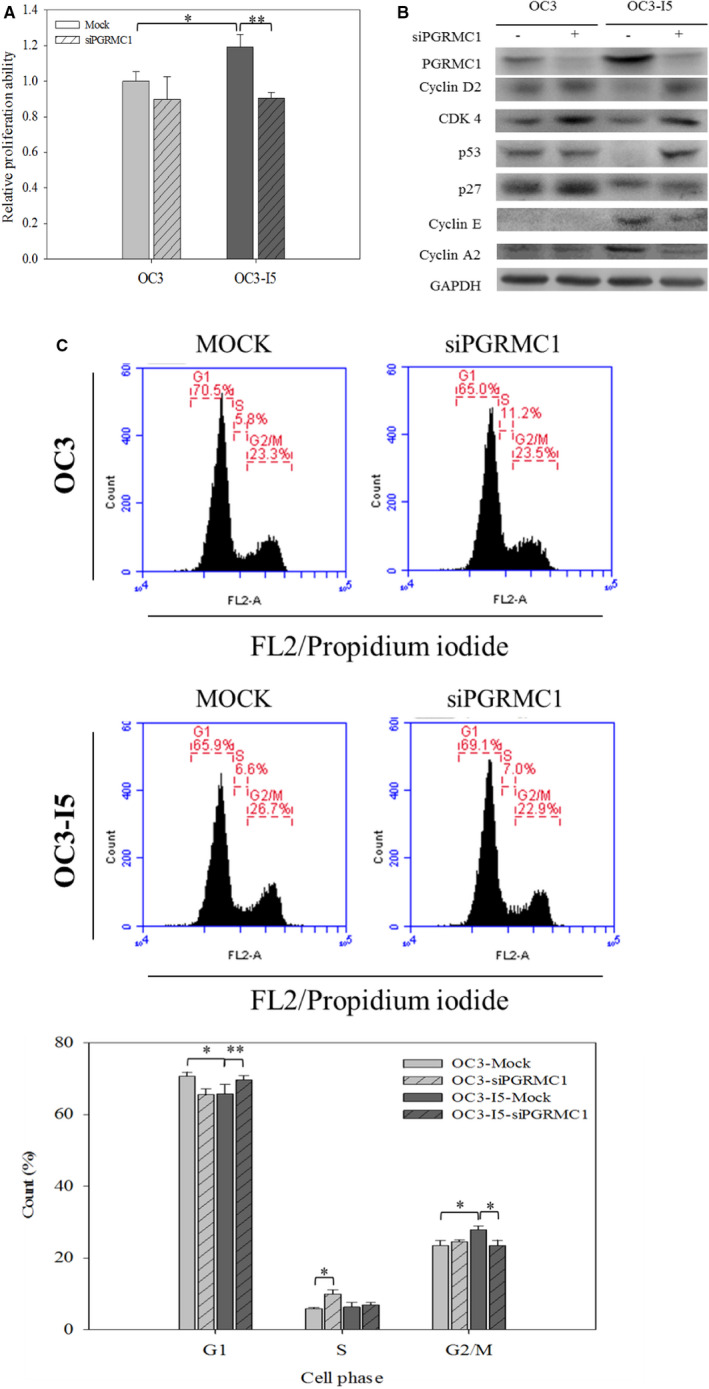
The effects of siPGRMC1 silencing on oral cancer proliferation. (A) Mock and PGRMC1 knockdown cells were seeded into 96‐well dish for 48 hours, followed by incubation with MTT and addition of DMSO. The absorbance at 540 nm was measured. Error bars denote mean ± SEM (n = 4). *P < .05, **P < .01. (B) The expression levels of G1/S transition promoting proteins, G1/S arrest signalling proteins and E2Ftranscriptional targets were validated by immunoblotting. G1/S transition promoting proteins, cyclin D2 and CDK4, were up‐regulated by siPGRMC1 treatment; therefore, G1/S arrest signalling proteins, p53 and p27, were overexpressed. At the downstream of G1/S arrest signalling, the expression levels of E2Ftranscriptional targets, cyclin E and cyclin A2, were inhibited by p53 and p27 in siPGRMC1 knockdown OC3‐I5. GAPDH was used as loading control. (C) PGRMC1‐silenced OC3 and OC3‐I5 cells were incubated with propidium iodide. After 15 minutes incubation, cells were analysed by flow cytometry and DNA content was quantified. Cell count was represented *y*‐axis, and propidium iodide was represented on *x*‐axis. The cell‐cycle status was decided by DNA content, and G_l_, S and G_2_/M phases were calculated. The percentage of cell‐cycle status was quantified. Error bars denote mean ± SEM (n = 3). *P < .05, **P < .01

To investigate whether PGRMC1 knockdown interferes oral cancer proliferation ability and how PGRMC1 acts, we performed MTT assay. As shown in Figure 5A, OC3‐I5 cells showed a 1.2‐fold better proliferation ability than OC3 cells. Nevertheless, the proliferation ability of OC3‐I5 cells declined when cells were transfected with siPGRMC1. We therefore investigated whether OC3 cells and OC3‐I5 cells with siPGRMC1 arrested in G1 phase. We first exanimated the expression levels of cyclin D2 and CDK4 (Figure 5B). The cyclin D2‐CDK4 complexes release E2F from Rb and E2F can drive G1/S phase transition; however, the immunoblotting analysis showed that OC3‐I5 cells expressed lower level of cyclin D2 and CDK4, which did not match the cell‐cycle assay. Subsequently, the expression levels of p53 and p27 were validated. Cell‐cycle inhibitor p53 and p27 prevents the formation of cyclin D‐CDK4 complexes; thus, p53 and p27function as inhibitors of cell‐cycle progression at G1to S phase. As shown in Figure 5B, only OC3‐I5 cells had low expression of p53; OC3 cells and cells treated with siPGRMC1 expressed high level of cell‐cycle inhibitors. The expression levels of E2F downstream target gene, cyclin E and cyclin A, were evaluated. Cyclin E and cyclin A can trigger G1/S transition and regulate S phase, respectively. Corresponding with cell‐cycle assay, OC3 cells and cells treated with siPGRMC1 expressed low level of cyclin E and cyclin A, but OC3‐I5 cells had increased levels of both proteins. These results indicated that PGRMC1 not only modulated invasion and migration but also triggered the entry of cells into G2/M phase via p53 down‐regulation and promoted oral cancer proliferation.

Then, we performed cell‐cycle analysis using propidium iodide (PI) staining to analyse how PGRMC1 modulates cell proliferation. The results demonstrated that OC3 cells got more cells stuck in G1 phase but fewer cells in G2/M phase than OC3‐I5 cells, and OC5‐I5 cells with PGRMC1 knockdown reverted back to OC3 cell‐cycle status (Figure 5C).

### 3.6 PGRMC1 knockdown influences lung metastases

**FIGURE 6:**
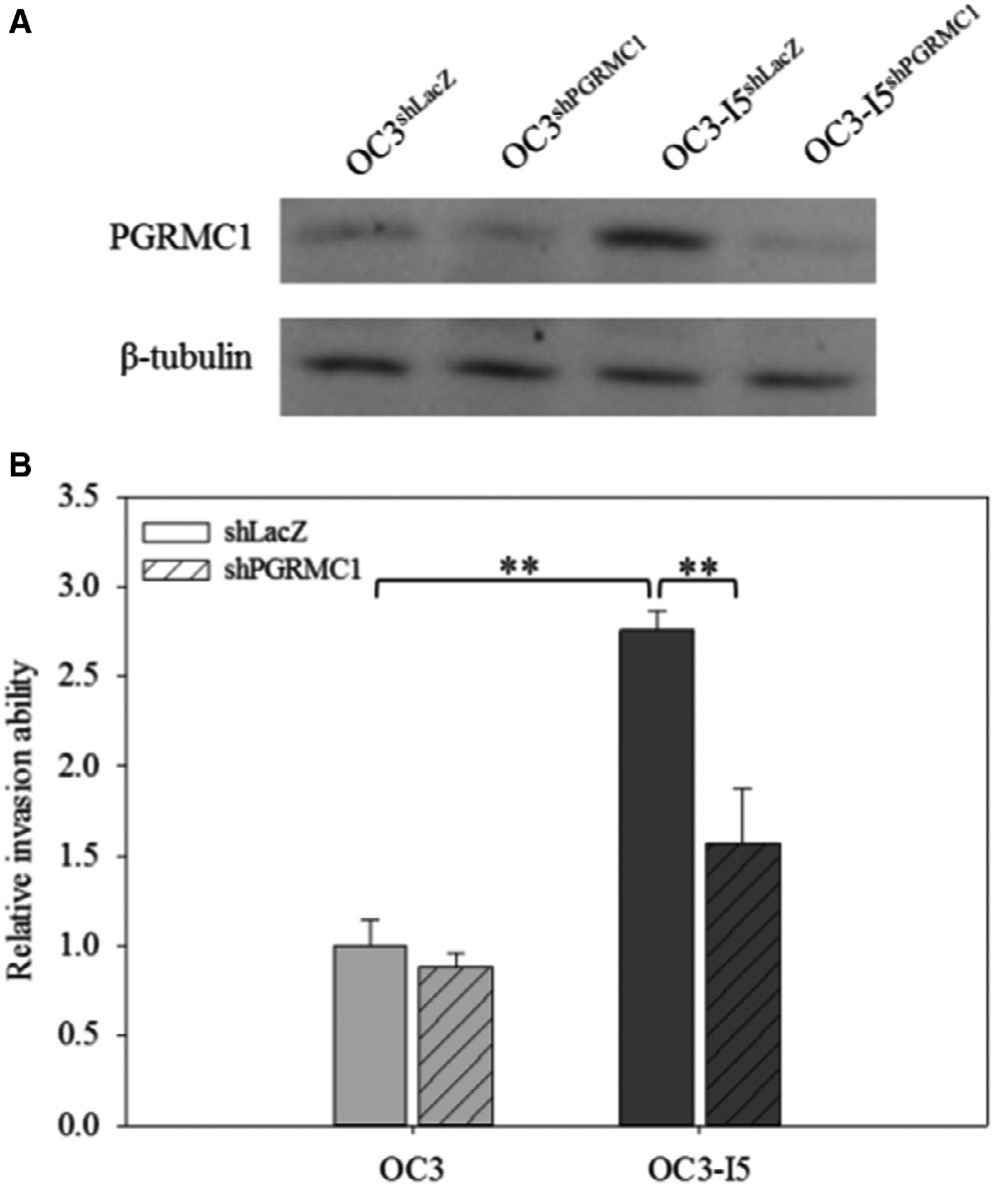
The invasion ability of OC3 and OC3‐I5 cells transfected with or without stable PGRMC1 knockdown. Cells were transfected with 10 nM shPGRMC1 or shLacZ (negative control). (A) The expression of PGRMC1 protein was detected by immunoblotting in OC3 and OC3 I‐5 cells with or without shPGRMC1 transfected. (B) Equal amount of cells (cells per insert) were seeded into matrix‐coated upper chamber of the insert with serum‐free media. After 18 hours, the invaded cells were fixed and stained by crystal violet. The crystal violet were dissolved in ethanol/acetic acid solution and quantified. Error bars denote mean ± SEM (n = 3). **P < .01

Due to the comprehensive effects of PGRMC1 on modulating oral cancer invasion, migration and proliferation, we examined the role of PGRMC1 in oral cancer invasion in vivo. shPGRMC1 plasmid DNA or shLacZ plasmid control was transfected in OC3 and OC3‐I5 cells, and the stable knockdown cell lines were established by puromycin selection. The puromycin selections were performed every two passages until the cell viability would not affected by puromycin. After stably knockdown cell line establishment, we performed immunoblotting to validate the PGRMC1 expression levels in OC3 and OC3‐I5 cells transfected with shPGRMC1 or shLacZ (Figure 6A), and the invasion ability of cells was also investigated before tumour implantation in mice (Figure 6B).

**FIGURE 7:**
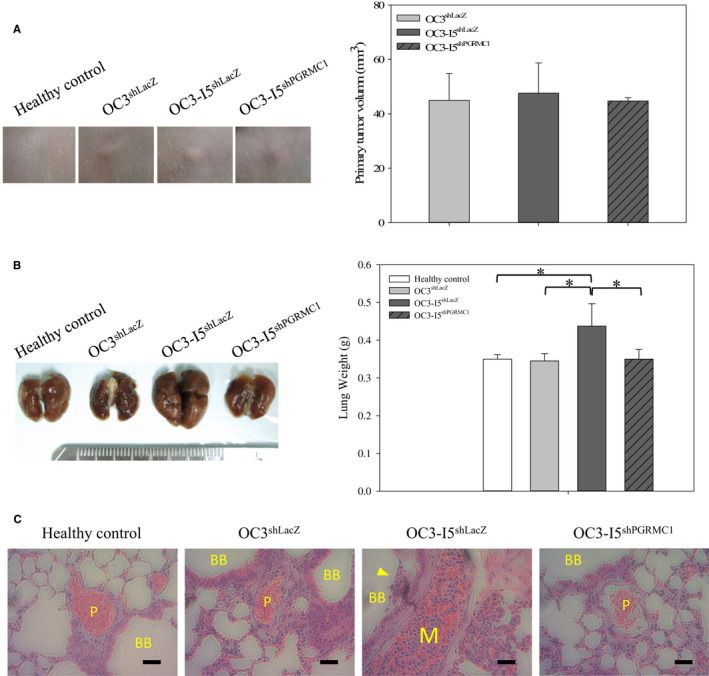
Stable knockdown of PGRMC1 in human oral cancer cells inhibits metastasis in vivo. (A) Nude mice were subcutaneously injected with OC3 and OC3‐I5 cells. On day 60 after cancer cell implantation, primary tumour volume measurements were performed. (B) On day 60 after cancer cell implantation, the mice were killed and the lungs were removed and fixed in 10% formalin. The weights of lungs were measured. Scale bar, 2 mm. (C) Haematoxylin and eosin (H&E) staining of OC3 and OC3‐I5 lung metastatic tumours. The extravasal oral cancer cells are indicated by yellow arrow. Symbol ‘P’ represents the pulmonary artery; symbol ‘M’ represents the metastasis in the pulmonary artery; and symbol ‘BB’ represents bronchial branch. Error bars denote mean ± SEM (n = 4). *P < .05. Bar represents 100 μm

5‐week‐old BALB/cAnN.Cg‐Foxnlnu/CrlNarl female mice were housed in a specific‐pathogen‐free environment. One week after housing, mice were injected with human oral cancer cells. 3 × 10^6^ OC3 and OC3‐I5 cells stably transfected with shPGRMC1 or shLacZ were mixed with Matrigel™ in PBS (1:7) and inoculated subcutaneously onto the right tight of mice (6 per group). After 60 days after implantation, the primary tumour sizes were measured (Figure 7A). The primary tumour growth rates were shown no difference between OC3^shLacZ^, OC3‐I5^shLacZ^ and OC3‐I5^shPGRMC1^ groups, which indicated that OC3 and OC3‐I5 cells had similar proliferation ability that were not influenced by PGRMC1 knockdown.

The mice were killed by zoletil/rompun 60 days after implantation, and the lungs were removed and fixed in 10% formalin. The weights of lungs were measured. Lungs of OC3‐I5^shLacZ^ group were heavier than healthy control, OC3^shLacZ^ and OC3‐I5^shPGRMC1^ groups (Figure 7B). These data suggested that OC3‐I5^shLacZ^ might had more severe lung metastasis than OC3^shLacZ^ group and the knockdown of PGRMC1 inhibited the cancer metastasis to lung. For further analysis, the lung samples were tissue trimming, tissue processing and embedding, section and H & E staining to examine tissue morphologies. The H & E staining of lung showed that metastasis in pulmonary artery and bronchial branch was only observed in OC3‐I5^shLacZ^ group but not in OC3‐I5^shPGRMC1^ group. Together, gross specimens and histological examination indicated that the knockdown of PGRMC1 can suppress distant metastasis of oral cancer cells in vivo.

## 4 DISCUSSION

A deeper insight into oral cancer metastasis mechanism is urgent to provide accurate diagnosis and therapy. Most of the oral cancer invasion studies are focused on transcriptional level. There are also some proteomics studies in this field. Several oral cancer metastasis–related protein candidates have been found, such as SOD2,^7^ SRC,^14^ tetranectin,^15^ PRDX4,^16^ PAHA2^16^ and caldesmon.^8^ However, most of these researches used clinical samples as material. Clinical samples exhibit different genetic backgrounds which may shadow the potential proteins. In our research, we used two oral cancer cell lines, OC3 and OC3‐I5, derived from a single patient to minimize the variation. An innovative, comprehensive and high‐resolution proteomic method based on 2D‐DIGE combining MALDI‐TOF/TOF MS analysis was performed in this research. The lysine‐labelling 2D‐DIGE analysis reveals that more than one hundred cytosolic proteins representing 91 individual proteins were differentially expressed and identified in invasive OC3‐I5 cells compared with OC3 cells. Numerous identified proteins were further validated by immunoblotting and ELISA assay using commercial primary antibodies. The quantifications of validation data were largely agreed with the 2D‐DIGE results.

The altered cytosolic proteins are mainly involved in cytoskeleton formation, protein degradation, protein folding, glycolysis, cell adhesion and extracellular matrix organization. These results suggested that to acquire invasion ability, oral cancer cells modulated themselves and microenvironment. Oral cancer cells modified their cytoskeletons to gain motility. The cytosolic proteins involved in protein degradation and folding indicated the change in protein biosynthesis and turnover. The glycolysis pathway seemed to be revised in order to provide enough energy. Outside the cells, proteins involved in proteolysis and extracellular matrix organization dedicated to reorganize the microenvironment for easier movement.

There were several identified potential proteins observed in oral cancer invasion. Most of the proteins were related to oxidative stress in invasive cancer. In cancer microenvironment, the nutrition supply by vascular is always poor, which results in regional hypoxia and acidic pH.^17^ Cancer with poorer environment would become more likely to metastasize. Under the limitation of nutrient and hypoxia, massive reactive oxygen species (ROS) are formation inside cells as a by‐product of increased signal cascades and metabolic activities. The modest level of ROS is quite important for invasive cancer cells as ROS can promote cell‐cycle progression, and activate receptor tyrosine kinases (RTK) and transcription factors that control invasion, angiogenesis and metastasis. On the other hand, excessive level of ROS damages DNA, RNA, proteins and other macromolecules, activates cell‐cycle inhibitor and induces apoptosis. Hence, the modulation of ROS and the fixation of oxidative damage are essential for invasive cancer cells. In our 2D results, several proteins related to ROS modulation had been discovered. Compared to OC3 cells, OC3‐I5 cells exhibit greater oxidative stress, which causes increment of damaged and toxic aldehydes and errors during protein synthesis. To limit the damages caused by ROS, aldose reductase, 26S protease regulatory subunit 6B and 26S proteasome non‐ATPase regulatory subunit 11 were overexpressed. Aldose reductase turns the ROS‐generated toxic aldehydes into inactive alcohols by NADPH cofactor and 26S protease regulatory subunits degrades the abnormal proteins to reduce endoplasmic reticulum (ER) stress.^18^ ROS‐catabolic protein, such as superoxide dismutase [Mn], mitochondrial (MnSOD) and peroxiredoxin 6 (Pxn6), were up‐regulated to decrease ROS level and recover cells from the oxidative damage.

Oxidative stress also elevates calcium concentration in invasive cells. The increase of calcium concentration would activate calcium‐dependent proteins such as protein kinase C (PKC), annexin A1 and L‐plastin in this study. Calcium promotes PKC membrane translocation and the binding of annexin A1 to PKC.^19^ The membrane translocation of PKC is regard as a marker of PKC activation, and the PKC membrane association is stabilized by the binding of annexin A1. PKC activation is regulated by 14‐3‐3 which is mentioned as PKC inhibitor, and the interaction between annexin A1 and PKC is inhibited by annexin A5. In our results, the expression level of annexin A1 was raised in invasive cells, while PKC inhibitor 14‐3‐3 and annexin A5 were decreased. This observation suggests PKC activation in invasive oral cancer cells.

The elevated calcium concentration and activated PKC promote L‐plastin binding to actin.^20^ L‐plastin is actin‐binding protein which modulates actin cytoskeleton. L‐plastin contains four actin‐binding domains and binds to actin filament during actin polymerization but do not bind to pre‐existing actin filament. The binding of L‐plastin to actin facilitates cell migration through promoting actin bundle formation that occurs mostly in cell protrusion. Beside, L‐plastin also stimulates the adhesion between cell and extracellular matrix via integrin‐mediated adhesion, which results in accelerating cell passing through extracellular matrix. L‐plastin binds actin in a calcium‐dependent manner and is regulated by PKC‐related phosphorylation at L‐Plastin serine 5. The previous studies showed that L‐plastin and its phosphorylation promoted cell invasion and migration in melanoma, colorectal and prostate carcinoma.^21^ However, whether it would regulate oral cancer invasion and how it regulates remain unknown. In our 2D results, the overexpressions of L‐plastin and integrin alpha‐6 were observed in invasive cells. These indicated that L‐plastin and integrin alpha‐6 might facilitate cell migration in a PKC‐dependent and calcium‐dependent manner.

There are still other proteins shown in our proteomic analysis, which have high potential in modulating oral cancer invasion. Anamorsin is also called cytokine‐induced apoptosis inhibitor 1 (CIAPIN1). CIAPIN1 is Ras signalling mediator and plays multiple roles in cancer development. CIAPIN1 is able to modulate multidrug resistance in breast, leukaemia and gastric cancer via regulating the expression of Bcl‐2, Bax and MDR2.^22^ Some previous studies demonstrated that CIAPIN1 has anti‐apoptosis effects and promotes cell migration and proliferation attributing to angiogenesis in vascular smooth muscle cells.^23^ Other studies suggested that the overexpression of CIAPIN1 would inhibit cell proliferation through attenuating cell cycle and promoting apoptosis.^24^ No matter whether CIAPIN1 performs apoptosis promoting or inhibiting effects, the apoptosis‐regulating property of CIAPIN1 is largely due to the character of post‐translational modification of cell‐cycle proteins, including cyclin D.^25^ There is only one research had mentioned the relationship between CIAPIN1 and metastasis. They found that CIAPIN1 is required for RhoGD12‐mediated invasion.^26^ CIAPIN1 was found to be up‐regulated in invasive oral cancer in our study, and the role of CIAPIN1 in oral cancer invasion remains unknown.

Guanine deaminase (GDA) has enzymatic effect on guanine catabolism. The overexpression of GDA in invasive oral cancer results in accelerating DNA turnover. This change might stimulate purine salvage pathway activity. Purine salvage pathway provides an economic and efficient pathway to synthesize purine, and allow cancer cells develop more rapidly.^27^ OC3‐I5 cells expressed higher level of GDA than OC3 cells, which indicate that OC3‐I5 cells have faster DNA turnover rate and develop rapidly than OC3 cells.

cAMP‐dependent protein kinase catalytic subunit PRKX (PRKX) is regulated by cAMP and mediated cAMP signalling. PRKX is sequence homologous and functional similar to protein kinase A (PKA).^28^ As long as PRKX binding to cAMP, PRKX phosphorylates its downstream targets which may include cAMP response element‐binding protein (CREB),^29^ mothers against decapentaplegic homolog 6 (Smad6),^30^ polycystin‐1 (PKD1)^31^ and histone; thus, PRKX regulate gene transcription and signal cascades. PRKX has multiple functions in cellular differentiation and epithelial morphogenesis. PRKX is shown to involve in angiogenesis through stimulating cell proliferation and migration, and forming vascular structure.^32^ In our research, PRKX is overexpressed in invasive OC3‐I5 cells compared to OC3 cells, which imply that PRKX may promote oral cancer migration and proliferation via cAMP signalling in invasive oral cancer cells.

PGRMC1, a haem‐binding protein, interacts with P450 family proteins and participates in sterol synthesis. In addition, two researches have demonstrated individually that PGRMC1^33^ and P450 family proteins^34^ are induced by carcinogen 2,3,7,8‐Tetrachlorodibenzodioxin (TCDD), which suggest P450s and PGRMC1 might in a upstream‐downstream relationship. PGRMC1 overexpresses in several types of tumours than in healthy tissues, such as breast, lung, ovary and colon cancer. Moreover, numerous researches have shown that PGRMC1 is overexpressed in chemo‐resistant tumour cells, such as doxorubicin‐resistant and camptothecin‐resistant breast cancer cells, cisplatin‐resistant ovarian cancer cells and doxorubicin‐resistant uterine cancer cells.^35^ Doxorubicin, camptothecin and cisplatin cause DNA damage and trigger apoptosis. PGRMC1 is induced as a consequence of DNA damage and protects cells from damage‐induced apoptosis. Inadvanced stage of breast^36^ and ovarian^37^ tumours, PGRMC1 expression level increased compared to early‐stage tumours. This phenomenon is only observed in clinical tumour specimens. Nevertheless, the roles of PGRMC1 in invasion and metastasis remain largely unknown, and there are no literature reports of the role of PGRMC1 in cancer invasion as we knew.

Our results demonstrated that PGRMC1 is crucial for oral cancer invasion and motility. Proteins such as CYP2J2, Src and FAK signalling pathways and the migration effectors JAM‐A and vinculin are involved in PGRMC1‐modulating cell invasion and migration. EMT elongation factors Twist and SIP1 are also participated in PGRMC1‐modulating cell invasion. Moreover, EMT elongation factor Snai1 and metalloproteinase 13 (MMP13) are down‐regulated by PGRMC1‐siRNA knockdown. The knockdown of PGRMC1 also attenuates cell proliferation of invasive oral cancer cells and p53‐mediated G1 arrest was observed in PGRMC1 knockdown OC3‐I5 cells. These observations suggested that PGRMC1 promotes cell proliferation through regulating p53 in invasive oral cancer. The cell cycle gene expression regulation via progesterone and PGRMC1 has been reported at transcriptional level,^38^ which can correspond to our results. Furthermore, the in vivo data suggested that PGRMC1 influence not only migration and invasion, but also metastasis in mouse model.

PGRMC1 has several binding partners, such as P450s, PAIR‐BP1, insig‐1 and SCAP, and modulates sterol synthesis, apoptosis suppression, cholesterol regulation via these binding partners.^35^ Among these binding partners, plasminogen activator inhibitor RNA‐binding protein 1 (PAIR‐BP1) might be an essential component for PGRMC1‐mediated oral cancer invasion. PAIR‐BP1 has a C‐terminal RNA‐binding domain, indicating the role in post‐transcriptional modification of mRNAs, especially the mRNA class participating in extracellular proteases regulation.^39^ Furthermore, PGRMC1 can be a regulator of gene expression.^40^ PGRMC1 was found not only in cytoplasm, but also within nucleus where it modulates gene expression pattern and affects de novo RNA and protein synthesis.^41^ Several studies revealed the gene expression pattern change causing by PGRMC1depletion; however, these studies focused particularly on cell death and survival‐related genes but no others.^41^, ^42^ In invasive oral cancer, knockdown of PGRMC1 affects lots of protein expression levels, such as p53, Twist, Snail and so on. PGRMC1 might modulate protein synthesis through mRNA modification via binding with PAIR‐BP1 or through transcriptional regulation via translocating into nucleus to upgrade the invasion, migration and proliferation abilities of oral cancer.

In this study, we performed lysine‐labelling 2D‐DIGE and MALDI‐TOF MS analysis for the identification of invasion‐related proteins in oral cancer. These altered proteins promoted cell migration, proliferation and anti‐apoptosis inside the cell. We classified and arranged the proteins identified in this study and proposed invasion mechanisms that may occur in OC3‐I5 cells. The identified up‐regulated proteins might serve as indicators for predicting the invasion ability of oral cancer, while the identified altered cytosolic proteins might serve as therapeutic targets for oral cancer patients after surgery to decrease incidence of cancer recurrence.

## CONFLICT OF INTERESTS

The authors declare that they have no conflict of interests.

## AUTHOR CONTRIBUTION

Ying‐Ray Lee and Hong‐Lin Chan involved in investigation. Hsun‐Yu Huang, Hsiu‐Chuan Chou, Ching‐Hsuan Law, Wan‐Ting Chang, Tzu‐Ning Wen, En‐Chi Liao and Kui‐Thong Tan involved in data curation. Meng‐Wei Lin, Li‐Hsun Lin, Yu‐Shan Wei, Yi‐Ting Tsai and Hsin‐Yi Chen involved in formal analysis. Wen‐Hung Kuo, Mei‐Lan Ko and Shing‐Jyh Chang involved in review and editing.

## Supporting information

Table S1

Fig S1

Fig S2‐1

Fig S2‐2

Fig S3

## Data Availability

The data that support the findings of this study are available from the corresponding author upon reasonable request.
