# Supplementary material for: Progesterone receptor membrane component 1 is involved in oral cancer cell metastasis
Source: J Cell Mol Med. 2020 Jul 16;24(17):9737–51. doi: 10.1111/jcmm.15535 (PMC7520311; doi:10.1111/jcmm.15535)
Supplement: Supplementary file 1 — Table S1 [file JCMM-24-9737-s001.docx]

Table 1

List of identified differentially expressed cytosolic proteins of OC3 and OC3-I5 cells. *MS/MS

| Spot No. | Swiss-prot No. | Protein name | MW | pI | No. matched peptides | Score | Cov. | Representative matched peptides | OC3-I5 / OC3 | Functional classification | Subcellular location |
| --- | --- | --- | --- | --- | --- | --- | --- | --- | --- | --- | --- |
| 1244 | P31947 | 14-3-3 protein sigma | 27871 | 4.68 | 18/54 | 160/56 | 54% | K.SNEEGSEEKGPEVR.E K.GEELSCEER.N | -1.62 | Singnal transduction | Cytoplasm |
| 668 | P43686 | 26S protease regulatory subunit 6B | 47451 | 5.09 | 11/42 | 101/56 | 19% | K.RFDAQTGADR.E R.ADTLDPALLRPGR.L | 1.39 | Protein degradation | Cytoplasm |
| 701 | O00231 | 26S proteasome non-ATPase regulatory subunit 11 | 47719 | 6.08 | 14/48 | 94/56 | 26% | R.YQEALHLGSQLLR.E K.TYHALSNLPK.A | 3.71 | Protein degradation | Cytoplasm |
| 224 | P11021 | 78 kDa glucose-regulated protein | 72402 | 5.07 | 9/11 | 126/56 | 0.16 | K.VTHAVVTVPAYFNDAQR.Q R.IINEPTAAAIAYGLDKR.E | -1.31 | Protein folding | ER |
| 1113 | P63261 | Actin, cytoplasmic 2 | 42108 | 5.31 | 8/63 | 93/56 | 28% | R.VAPEEHPVLLTEAPLNPK.A K.SYELPDGQVITIGNER.F | 32.90 | Cytoskeleton | Cytoplasm |
| 389 | P47895 | Aldehyde dehydrogenase family 1 member A3 | 56871 | 6.99 | 9/20 | 105/56 | 15% | K.IFINNEWHESK.S K.TIPTDDNVVCFTR.H | -1.39 | Redox regulation | Cytoplasm |
| 517 | P05091 | Aldehyde dehydrogenase, mitochondrial | 56859 | 6.63 | 9/31 | 79/56 | 18% | K.TIPIDGDFFSYTR.H R.TFVQEDIYDEFVER.S | -1.45 | Redox regulation | Mitochondria |
| 1048 | P15121 | Aldose reductase | 36230 | 6.51 | 6/23 | 71/56 | 15% | K.REELFIVSK.L K.LWCTYHEK.G | 1.74 | Redox regulation | Cytoplasm |
| 599 | P06733 | Alpha-enolase | 47481 | 7.01 | 17/67 | 131/56 | 38% | R.AAVPSGASTGIYEALELR.D R.IGAEVYHNLK.N | 1.43 | Glycolysis | Cytoplasm |
| 622 | P06733 | Alpha-enolase | 47481 | 7.01 | 7/24 | 79/56 | 10% | R.IGAEVYHNLK.N K.YNQLLR.I | 1.54 | Glycolysis | Cytoplasm |
| 1778 | P06733 | Alpha-enolase | 47481 | 7.01 | 11/35 | 108/56 | 24% | K.LAMQEFMILPVGAANFR.E K.LAQANGWGVMVSHR.S | 1.43 | Glycolysis | Cytoplasm |
| 1779 | P06733 | Alpha-enolase | 47481 | 7.01 | 11/26 | 122/56 | 26% | R.IGAEVYHNLK.N K.YNQLLR.I | 1.48 | Glycolysis | Cytoplasm |
| 860 | Q6FI81 | Anamorsin | 34131 | 5.44 | 6/26 | 91/56 | 23% | R.ILRPGGCLFLK.E R.EPLTPEEVQSVR.E | 3.62 | Anti-apoptosis | Cytoplasm |
| 254 | Q86XL3 | Ankyrin repeat and LEM domain-containing protein 2 | 104905 | 6.66 | 10/29 | 78/56 | 11% | K.AGLKCGPITSTTR.F K.KLAQALLEQGGR.L | -1.39 | Cell cycle | ER |
| 1083 | P04083 | Annexin A1 | 38918 | 6.57 | 13/35 | 134/56 | 44% | R.KGTDVNVFNTILTTR.S K.CATSKPAFFAEK.L | 1.33 | Calcium signaling / Calcium/phospholipid-binding | Plasma membrane |
| 1029* | P07355 | Annexin A2 | 38808 | 7.57 | 15/53 | 186/56 | 46% | K.GLGTDEDSLIEIICSR.T K.DIISDTSGDFR.K | -1.33 | Calcium signaling / Calcium/phospholipid-binding | Secreted |
| 1151 | P12429 | Annexin A3 | 36524 | 5.63 | 16/51 | 136/56 | 37% | K.SLGDDISSETSGDFRK.A K.ALLTLADGRR.D | -1.51 | Calcium signaling / Calcium/phospholipid-binding | Plasma membrane |
| 1110 | P09525 | Annexin A4 | 36088 | 5.84 | 10/19 | 106/56 | 27% | R.VLVSLSAGGR.D R.DEGNYLDDALVR.Q | 2.08 | Calcium signaling / Calcium/phospholipid-binding | Plasma membrane |
| 1125 | Q5T1B0 | Axonemal dynein light chain domain-containing protein 1 | 118637 | 5.49 | 11/26 | 86/56 | 13% | R.DIWSSATYELALKVIER.N K.EYEIRINGDNGYSK.I | 2.19 | Unknown | Unknown |
| 704 | Q6NUK1 | Calcium-binding mitochondrial carrier protein SCaMC-1 | 53548 | 6.00 | 8/28 | 79/56 | 13% | R.QLLAGGIAGAVSR.T K.MNIFGGFRQMVK.E | 5.72 | Transport | Mitochondria |
| 347 | P27797 | Calreticulin | 48283 | 4.29 | 15/64 | 104/56 | 21% | K.HEQNIDCGGGYVK.L K.EQFLDGDGWTSR.W | 1.52 | Calcium signaling / Protein folding | ER |
| 706 | P51817 | cAMP-dependent protein kinase catalytic subunit PRKX | 41041 | 6.36 | 6/43 | 66/56 | 12% | K.VMSIPDVIRLK.Q K.VMSIPDVIR.L | 10.68 | Singnal transduction | Cytoplasm |
| 1225 | P07858 | Cathepsin B | 38766 | 5.88 | 6/46 | 80/56 | 22% | K.HYGYNSYSVSNSEK.D K.ICEPGYSPTYK.Q | 1.44 | Protein degradation | Lysosome |
| 1242 | P07339 | Cathepsin D | 45037 | 6.10 | 10/28 | 105/56 | 15% | K.QPGITFIAAK.F K.FDGILGMAYPR.I | 1.74 | Protein degradation | Lysosome |
| 1191 | P08962 | CD63 antigen | 26474 | 8.14 | 8/33 | 68/56 | 0.21 | K.VMSEFNNNFRQQMENYPK.N R.MQADFKCCGAANYTDWEK.I | -1.34 | Growth regulation | Plasma membrane |
| 1276 | Q9Y696 | Chloride intracellular channel protein 4 | 28982 | 5.45 | 8/19 | 75/56 | 28% | K.AGSDGESIGNCPFSQR.L K.HPESNTAGMDIFAK.F | 1.49 | Transport | Plasma membrane |
| 1592 | P23528 | Cofilin-1 | 18719 | 8.22 | 10/29 | 123/56 | 60% | K.AVLFCLSEDKK.N R.YALYDATYETK.E | 2.44 | Cytoskeleton regulation | Cytoplasm |
| 700 | P45452 | Collagenase 3 | 54014 | 5.32 | 8/43 | 73/56 | 21% | K.VWSDVTPLNFTR.L R.LHPQQVDAELFLTK.S | 9.82 | Extracellular matrix degradation | Secreted |
| 872 | P51589 | Cytochrome P450 2J2 | 57859 | 8.76 | 7/22 | 65/56 | 14% | R.FTLTALRNFGLGK.K K.LKLFVSHMIDK.H | 70.90 | Redox regulation | ER |
| 172 | Q9NYF0 | Dapper homolog 1 | 91145 | 8.93 | 10/24 | 71/56 | 10% | K.YQCDLVSK.N K.THPVRTNKPR.T | 1.85 | Singnal transduction | Cytoplasm |
| 504 | O60260 | E3 ubiquitin-protein ligase parkin | 53602 | 6.71 | 8/32 | 65/56 | 16% | K.LRVQCSTCR.Q K.CGAHPTSDK.E | -1.40 | Protein degradation | Cytoplasm |
| 863 | Q93070 | Ecto-ADP-ribosyltransferase 4 | 36197 | 9.31 | 6/22 | 74/56 | 20% | R.TPQQYERSFHFK.Y K.DVHFNAYTGATIR.F | 4.44 | Biosynthesis | Plasma membrane |
| 1187 | Q96C19 | EF-hand domain-containing protein D2 | 26795 | 5.15 | 12/47 | 106/56 | 43% | R.ADLNQGIGEPQSPSRR.V R.RVFNPYTEFK.E | 1.68 | Anti-apoptosis | Plasma membrane |
| 1060 | P29692 | Elongation factor 1-delta | 31217 | 4.90 | 7/24 | 73/56 | 22% | R.QENGASVILR.D R.IASLEVENQSLR.G | 1.73 | Protein synthesis | Cytoplasm |
| 1062 | P29692 | Elongation factor 1-delta | 31217 | 4.90 | 8/34 | 109/56 | 29% | R.IASLEVENQSLR.G K.LVPVGYGIR.K | -1.34 | Protein synthesis | Cytoplasm |
| 1065* | P29692 | Elongation factor 1-delta | 31217 | 4.90 | 1 | 69/56 | 3% | K.LVPVGYGIR.K | -1.37 | Protein synthesis | Cytoplasm |
| 1327* | P56537 | Eukaryotic translation initiation factor 6 | 27095 | 4.56 | 1 | 73/56 | 4% | R.NSLPDTVQIR.R | -1.38 | Protein synthesis | Cytoplasm |
| 400 | Q8IYI6 | Exocyst complex component 8 | 82432 | 5.35 | 7/18 | 66/56 | 9% | R.QLESGGFEAR.L K.ESLSTAAECVK.V | -1.65 | Exocytosis | Cytoplasm |
| 1732 | P09382 | Galectin-1 | 15048 | 5.34 | 8/77 | 100/56 | 57% | R.FNAHGDANTIVCNSK.D K.DGGAWGTEQR.E | 1.51 | Growth regulation | Secreted |
| 716 | O60547 | GDP-mannose 4,6 dehydratase | 42265 | 6.87 | 8/38 | 70/56 | 21% | R.CPSARGSGDGEMGKPR.N K.TIVWEGKNENEVGR.C | 6.63 | Biosynthesis | Cytoplasm |
| 1128 | P14136 | Glial fibrillary acidic protein | 49907 | 5.42 | 11/45 | 76/56 | 24% | K.LQDETNLRLEAENNLAAYR.Q R.KIESLEEEIR.F | 3.04 | Cytoskeleton | Cytoplasm |
| 441 | P11413 | Glucose-6-phosphate 1-dehydrogenase | 59675 | 6.39 | 7/19 | 74/56 | 14% | R.DGLLPENTFIVGYAR.S R.NSYVAGQYDDAASYQR.L | -1.50 | Glycolysis | Cytoplasm |
| 575 | P48637 | Glutathione synthetase | 52523 | 5.67 | 13/33 | 116/56 | 25% | K.QIEINTISASFGGLASR.T R.AIENELLAR.N | -1.30 | Biosynthesis | Cytoplasm |
| 699 | Q9Y2T3 | Guanine deaminase | 51484 | 5.44 | 8/37 | 90/56 | 17% | K.VCMDLNDTFPEYK.E R.DLHIQSHISENR.D | 2.86 | Catabolism | Cytoplasm |
| 249 | P08107 | Heat shock 70 kDa protein 1A/1B | 70294 | 5.48 | 13/35 | 105/56 | 21% | R.IINEPTAAAIAYGLDR.T K.ATAGDTHLGGEDFDNR.L | -1.34 | Protein folding | Cytoplasm |
| 5 | P34932 | Heat shock 70 kDa protein 4 | 95127 | 5.11 | 9/30 | 84/56 | 11% | K.VLATAFDTTLGGR.K K.EDIYAVEIVGGATR.I | 1.51 | Protein folding | Cytoplasm |
| 1352 | P04792 | Heat shock protein beta-1 | 22826 | 5.98 | 6/16 | 88/56 | 25% | R.LFDQAFGLPR.L R.QDEHGYISR.C | -2.39 | Protein folding | Cytoplasm |
| 1432 | [Q9Y5Z4](http://www.uniprot.org/uniprot/Q9Y5Z4) | Heme-binding protein 2 | 22861 | 4.58 | 6/28 | 85/56 | 0.29 | R.SFDGFSSAQK.N K.NQEQLLTLASILR.E | -1.80 | Necrosis | Cytoplasm |
| 448 | Q6ZVN8 | Hemojuvelin | 45964 | 7.57 | 7/48 | 73/56 | 17% | R.GGGVGSGGLCR.A R.SFHHHFHTCR.V | -1.30 | Iron homeostasis | Plasma membrane |
| 1114* | P22626 | Heterogeneous nuclear ribonucleoproteins A2/B1 | 37464 | 8.97 | 1 | 81/56 | 4% | R.GGGGNFGPGPGSNFR.G | 1.36 | Gene regulation | Nucleus |
| 547 | P12081 | Histidyl-tRNA synthetase, cytoplasmic | 57944 | 5.72 | 10/27 | 78/56 | 16% | K.LKAQLGPDESK.Q K.VPCVGLSIGVER.I | -1.40 | Protein synthesis | Cytoplasm |
| 7 | P23229 | Integrin alpha-6 | 127751 | 6.39 | 10/18 | 112/56 | 9% | R.TGGLYSCDITAR.G K.LIATFPDTLTYSAYR.E | 1.35 | Cell adhesion | Plasma membrane |
| 876 | P01570 | Interferon alpha-14 | 22390 | 6.82 | 9/34 | 76/56 | 25% | R.RISPFSCLK.D K.KYSPCAWEVVR.A | 26.84 | Immuno response | Secreted |
| 662 | Q6TDP4 | Kelch-like protein 17 | 70628 | 7.64 | 6/13 | 68/56 | 10% | K.AAHRYVLQHFVDVAK.T R.VGVAAVGNR.L | -5.05 | Cell junction | Plasma membrane |
| 167 | P13645 | Keratin, type I cytoskeletal 10 | 59020 | 5.13 | 12/28 | 118/56 | 18% | R.LASYLDKVR.A K.HGNSHQGEPR.D | 2.25 | Cytoskeleton | Cytoplasm |
| 855 | P13645 | Keratin, type I cytoskeletal 10 | 59020 | 5.13 | 17/38 | 151/56 | 23% | K.HGNSHQGEPR.D R.SQYEQLAEQNRK.D | 5.23 | Cytoskeleton | Cytoplasm |
| 886 | P13645 | Keratin, type I cytoskeletal 10 | 59020 | 5.13 | 15/52 | 103/56 | 21% | R.LKYENEVALR.Q R.QSVEADINGLRR.V | 10.28 | Cytoskeleton | Cytoplasm |
| 586 | P02533 | Keratin, type I cytoskeletal 14 | 51872 | 5.09 | 14/32 | 125/56 | 26% | R.ALEEANADLEVK.I K.TRLEQEIATYR.R | -1.73 | Cytoskeleton | Cytoplasm |
| 659 | Q04695 | Keratin, type I cytoskeletal 17 | 48361 | 4.97 | 20/56 | 178/56 | 36% | R.LSVEADINGLRR.V R.KDAEDWFFSK.T | -5.25 | Cytoskeleton | Cytoplasm |
| 675 | P05783 | Keratin, type I cytoskeletal 18 | 48029 | 5.34 | 10/36 | 93/56 | 17% | R.AQIFANTVDNAR.I R.QSVENDIHGLR.K | 1.83 | Cytoskeleton | Cytoplasm |
| 688 | P05783 | Keratin, type I cytoskeletal 18 | 48029 | 5.34 | 21/44 | 197/56 | 33% | R.AQIFANTVDNAR.I R.SLETENRR.L | -1.36 | Cytoskeleton | Cytoplasm |
| 659 | P35527 | Keratin, type I cytoskeletal 9 | 62255 | 5.14 | 7/28 | 85/56 | 13% | R.FSSSGGGGGGGR.F R.FSSSSGYGGGSSR.V | -5.25 | Cytoskeleton | Cytoplasm |
| 607 | P05787 | Keratin, type II cytoskeletal 8 | 53671 | 5.52 | 16/46 | 153/56 | 27% | R.QLETLGQEK.L R.LEGLTDEINFLR.Q | -1.46 | Cytoskeleton | Cytoplasm |
| 1767 | P05787 | Keratin, type II cytoskeletal 8 | 53671 | 5.52 | 15/43 | 144/56 | 26% | R.LEGLTDEINFLR.Q R.SLDMDSIIAEVK.A | 2.63 | Cytoskeleton | Cytoplasm |
| 693 | Q9NSK0 | Kinesin light chain 4 | 69054 | 5.82 | 13/48 | 83/56 | 20% | K.NNLASCYLK.Q K.YAEAETLYKEILTR.A | 1.98 | Vesicle trafficking | Cytoplasm |
| 1107 | P00338 | L-lactate dehydrogenase A chain | 36950 | 8.44 | 11/38 | 127/56 | 27% | R.VIGSGCNLDSAR.F K.FIIPNVVK.Y | 1.30 | Glycolysis | Cytoplasm |
| 1738 | P61626 | Lysozyme C | 16982 | 9.38 | 6/38 | 79/56 | 32% | R.STDYGIFQINSR.Y R.ATNYNAGDR.S | -1.34 | Immuno response | Secreted |
| 1756 | P14174 | Macrophage migration inhibitory factor | 12639 | 7.74 | 6/31 | 67/56 | 29% | K.LLCGLLAER.L M.PMFIVNTNVPR.A | -1.46 | Immuno response | Secreted |
| 1460 | O00264 | Membrane-associated progesterone receptor component 1 | 21772 | 4.56 | 8/32 | 91/56 | 28% | R.ILMAINGKVFDVTK.G K.FYGPEGPYGVFAGR.D | 2.39 | Singnal transduction | Microsome |
| 653 | O75439 | Mitochondrial-processing peptidase subunit beta | 55073 | 6.38 | 9/27 | 85/56 | 15% | R.STQAATQVVLNVPETR.V K.FHFGDSLCTHK.G | -2.03 | Protein degradation | Mitochondria |
| 431 | Q9NTG7 | NAD-dependent protein deacetylase sirtuin-3, mitochondrial | 43945 | 8.98 | 6/18 | 69/56 | 14% | M.AFWGWR.A R.LVLGGRDDVSAGLR.G | -1.73 | Cellular energy metabolism | Mitochondria |
| 722 | Q9NXR1 | Nuclear distribution protein nudE homolog 1 | 38842 | 5.20 | 7/41 | 73/56 | 21% | R.QELAVQQKQEKPR.T K.QEKPRTPMPSSVEAER.T | 30.45 | Growth regulation | Cytoplasm |
| 1792 | P20472 | Parvalbumin alpha | 12051 | 4.98 | 7/25 | 85/56 | 38% | K.ETKMLMAAGDK.D K.MLMAAGDKDGDGK.I | -1.43 | Calcium regulation / Cell motility | Nucleus |
| 1337 | P30041 | Peroxiredoxin-6 | 25133 | 6.00 | 8/52 | 92/56 | 33% | R.DFTPVCTTELGR.A R.NRELAILLGMLDPAEK.D | 1.39 | Redox regulation | Cytoplasm |
| 322 | P13796 | Plastin-2 | 70814 | 5.29 | 15/29 | 170/56 | 22% | K.AYYHLLEQVAPK.G R.NWMNSLGVNPR.V | 1.42 | Cytoskeleton regulation | Cytoplasm |
| 311 | Q8WUM4 | Programmed cell death 6-interacting protein | 96590 | 6.13 | 12/39 | 73/56 | 14% | K.MVPVSVQQSLAAYNQRK.A K.SLLSNLDEVKK.E | 16.12 | Vesicle trafficking | Cytoplasm |
| 711 | Q9UQ80 | Proliferation-associated protein 2G4 | 44101 | 6.13 | 7/18 | 67/56 | 14% | K.AAHLCAEAALR.L K.EGEFVAQFK.F | 1.70 | Growth regulation | Cytoplasm |
| 1459 | P28072 | Proteasome subunit beta type-6 | 25570 | 4.80 | 6/23 | 76/56 | 19% | R.TTTGSYIANR.V R.LAAIAESGVER.Q | -1.33 | Protein degradation | Cytoplasm |
| 1143 | Q8TCP9 | Protein FAM200A | 66918 | 5.82 | 8/34 | 71/56 | 13% | K.HCKGISSDGTANMTGK.H K.EISPSLMDVLK.N | 22.99 | Unknown | Plasma membrane |
| 1106 | Q7L592 | Protein midA homolog, mitochondrial | 49435 | 8.47 | 8/26 | 79/56 | 12% | K.GYYVYRDMLGEK.G K.NMGIDVRLK.V | 1.55 | Electron transport | Mitochondria |
| 595 | O15297 | Protein phosphatase 1D | 67374 | 9.14 | 9/33 | 80/56 | 18% | R.DPLPDAGASPAPSRCCR.R R.IEGLGGSVMNK.S | 1.61 | Cell cycle | Nucleus |
| 857 | Q9H5N1 | Rab GTPase-binding effector protein 2 | 63960 | 4.76 | 7/19 | 71/56 | 17% | R.QQQQQDCEEKER.E R.EIVLPMEKEIEELK.A | 1.47 | Vesicle trafficking | Cytoplasm |
| 553 | Q92599 | Septin-8 | 56234 | 5.89 | 7/34 | 81/56 | 13% | R.FSNAEPEPR.S R.SLFDYHDTR.I | 1.56 | Vesicle trafficking | Cytoplasm |
| 1202 | O14830 | Serine/threonine-protein phosphatase with EF-hands 2 | 87433 | 6.70 | 7/17 | 78/56 | 12% | R.DFLTRIFTEDR.F K.LGPALTPHIVQYQANK.V | 1.96 | Phototransduction | Cytoplasm |
| 820 | P36952 | Serpin B5 | 42530 | 5.72 | 11/52 | 155/56 | 32% | K.DVPFGFQTVTSDVNK.L K.DELNADHPFIYIIR.H | -1.82 | Growth regulation | Secreted |
| 1409 | Q7Z614 | Sorting nexin-20 | 36668 | 8.94 | 6/19 | 61/56 | 16% | R.KHLTGNFAEEMICER.R K.DFVTLQERLEESQLR.R | -1.31 | Vesicle trafficking | Cytoplasm |
| 1445 | P04179 | Superoxide dismutase [Mn], mitochondrial | 24878 | 8.35 | 7/28 | 78/56 | 29% | K.HHAAYVNNLNVTEEK.Y K.GDVTAQIALQPALK.F | 2.69 | Redox regulation | Mitochondria |
| 420 | Q92609 | TBC1 domain family member 5 | 89519 | 6.10 | 10/30 | 71/56 | 13% | K.EIHITNPRK.V K.VSNSLINFGRK.L | -3.22 | Vesicle trafficking | Cytoplasm |
| 439 | P17987 | T-complex protein 1 subunit alpha | 60819 | 5.80 | 7/21 | 98/56 | 12% | K.IHPTSVISGYR.L K.IACLDFSLQK.T | -1.49 | Protein folding | Cytoplasm |
| 404 | P48643 | T-complex protein 1 subunit epsilon | 60089 | 5.45 | 7/21 | 77/56 | 11% | R.IADGYEQAAR.V K.IAILTCPFEPPKPK.T | -2.20 | Protein folding | Cytoplasm |
| 393 | Q99832 | T-complex protein 1 subunit eta | 59842 | 7.55 | 16/58 | 122/56 | 29% | K.QVKPYVEEGLHPQIIIR.A R.CQVFEETQIGGER.Y | -1.98 | Protein folding | Cytoplasm |
| 1231 | Q8IY51 | Tigger transposable element-derived protein 4 | 58115 | 6.05 | 10/47 | 73/56 | 20% | R.LRTAFYTDLEEALMR.W R.TAFYTDLEEALMRWYR.I | 1.76 | Gene regulation | Nucleus |
| 1183 | Q96FX7 | tRNA (adenine-N(1)-)-methyltransferase catalytic subunit TRMT61A | 31704 | 6.89 | 7/17 | 98/56 | 34% | R.HSVDLIGRPFGSKVTCGR.G R.FCSFSPCIEQVQR.T | 1.70 | Gene regulation | Nucleus |
| 1142 | P09493 | Tropomyosin alpha-1 chain | 32746 | 4.69 | 11/36 | 101/56 | 28% | K.KATDAEADVASLNR.R K.ATDAEADVASLNR.R | 1.33 | Cytoskeleton | Cytoplasm |
| 583 | Q13885 | Tubulin beta-2A chain | 50274 | 4.78 | 6/20 | 70/56 | 12% | R.AILVDLEPGTMDSVR.S R.FPGQLNADLR.K | -1.97 | Cytoskeleton | Cytoplasm |
| 432 | Q16222 | UDP-N-acetylhexosamine pyrophosphorylase | 59131 | 5.92 | 9/34 | 72/56 | 14% | K.VADPRFIGFCIQK.G K.KFVVYEVLR.E | -1.46 | Biosynthesis | Cytoplasm |
| 493 | P54727 | UV excision repair protein RAD23 homolog B | 43202 | 4.79 | 7/28 | 88/56 | 16% | K.DAFPVAGQK.L R.ASFNNPDR.A | 1.33 | DNA repair | Nucleus |
| 655 | P08670 | Vimentin | 53676 | 5.06 | 13/47 | 92/56 | 27% | K.VESLQEEIAFLKK.L R.ISLPLPNFSSLNLR.E | -1.79 | Cytoskeleton | Cytoplasm |
| 687 | P08670 | Vimentin | 53676 | 5.06 | 9/38 | 90/56 | 16% | K.FADLSEAANR.N K.VELQELNDR.F | 1.63 | Cytoskeleton | Cytoplasm |
| 689 | P08670 | Vimentin | 53676 | 5.06 | 9/31 | 77/56 | 16% | K.SKFADLSEAANR.N K.FADLSEAANRNNDALR.Q | 1.90 | Cytoskeleton | Cytoplasm |
| 727 | P08670 | Vimentin | 53676 | 5.06 | 11/36 | 81/56 | 23% | R.RMFGGPGTASRPSSSR.S R.TNEKVELQELNDR.F | 1.62 | Cytoskeleton | Cytoplasm |
| 398 | O43309 | Zinc finger and SCAN domain-containing protein 12 | 71888 | 6.28 | 9/30 | 75/56 | 15% | K.TSSKFENDMSK.S R.DKPYQCTQCNK.S | -1.84 | Gene regulation | Nucleus |
| 320 | Q68DI1 | Zinc finger protein 776 | 61285 | 8.68 | 7/38 | 74/56 | 13% | K.KLDDDANHHQDQK.Q K.LDDDANHHQDQK.Q | 32.61 | Gene regulation | Nucleus |
