# Supplementary figures and images for: Progesterone receptor membrane component 1 is involved in oral cancer cell metastasis
Source: J Cell Mol Med. 2020 Jul 16;24(17):9737–51. doi: 10.1111/jcmm.15535 (PMC7520311; doi:10.1111/jcmm.15535)

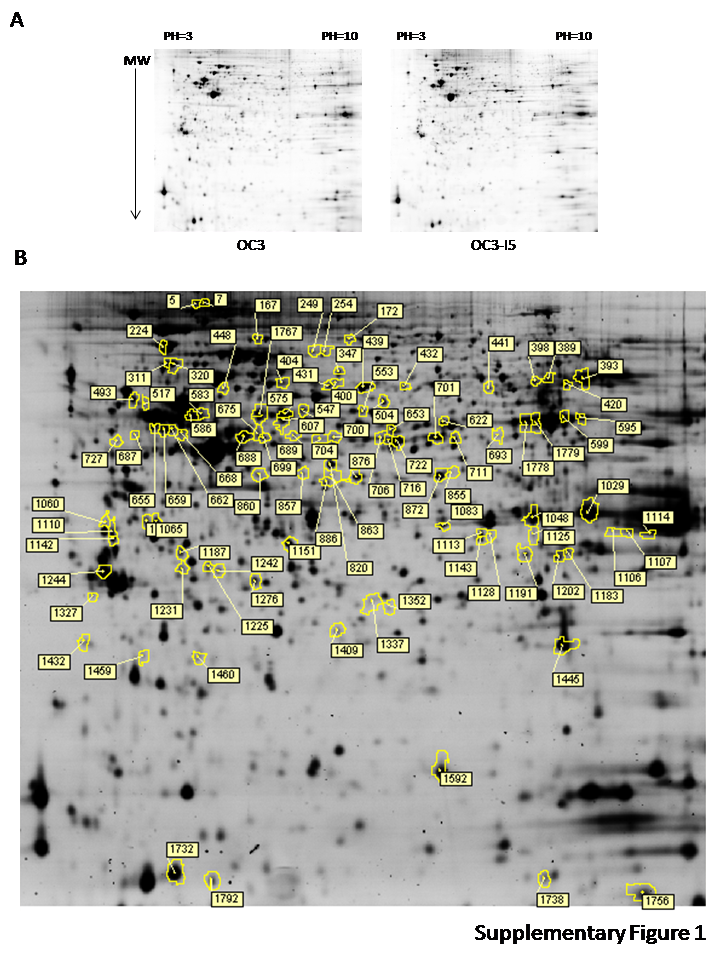

Supplement: Supplementary file 2 — Fig S1 [file JCMM-24-9737-s002.tif]

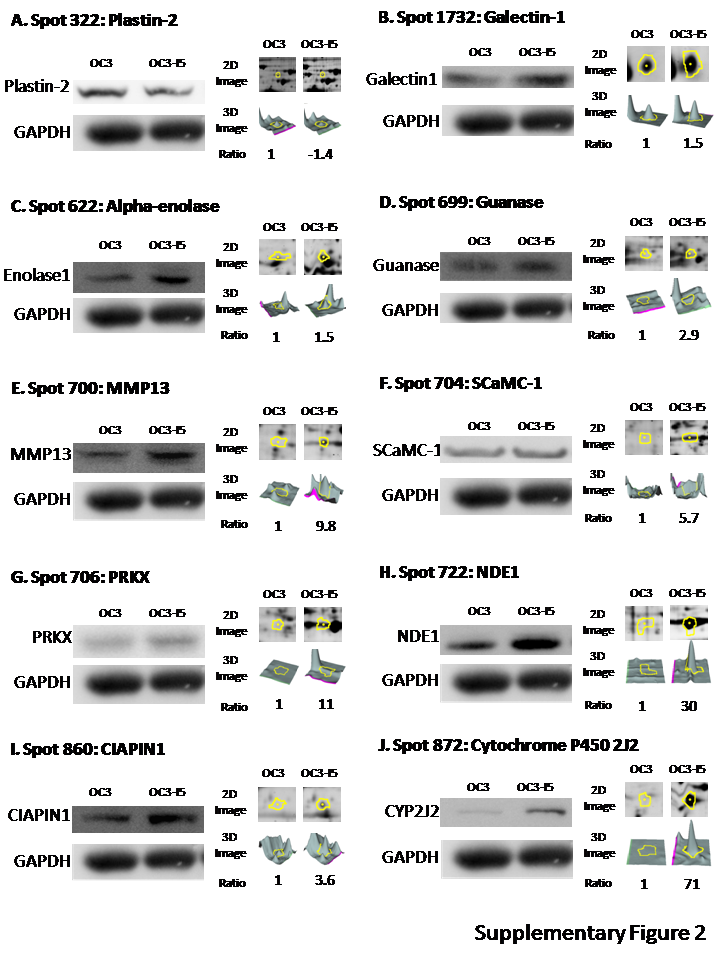

Supplement: Supplementary file 3 — Fig S2‐1 [file JCMM-24-9737-s003.TIF]

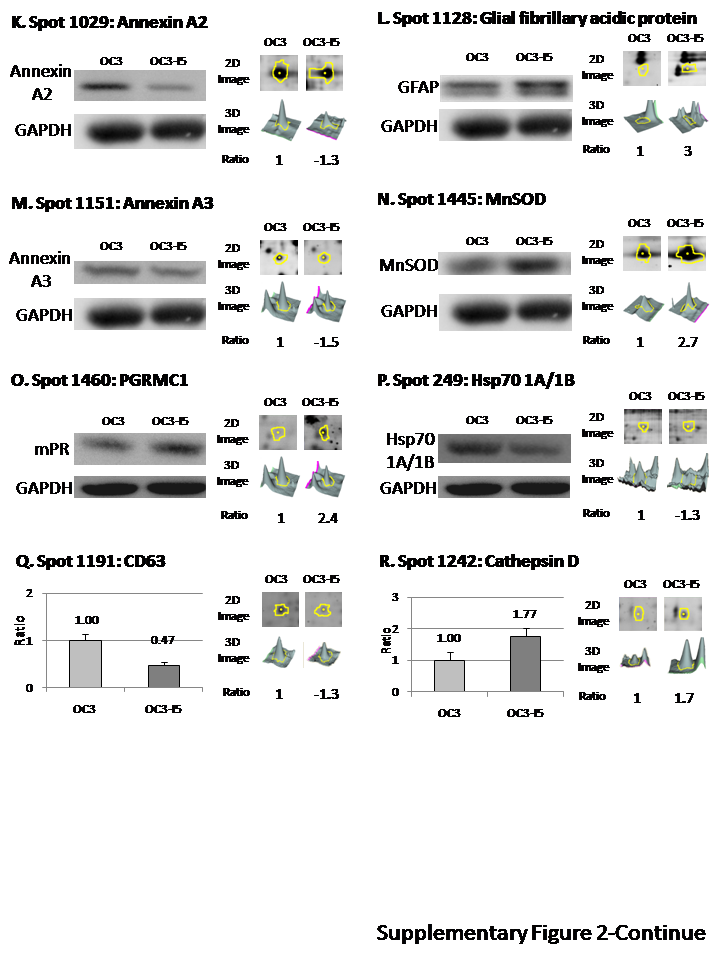

Supplement: Supplementary file 4 — Fig S2‐2 [file JCMM-24-9737-s004.TIF]

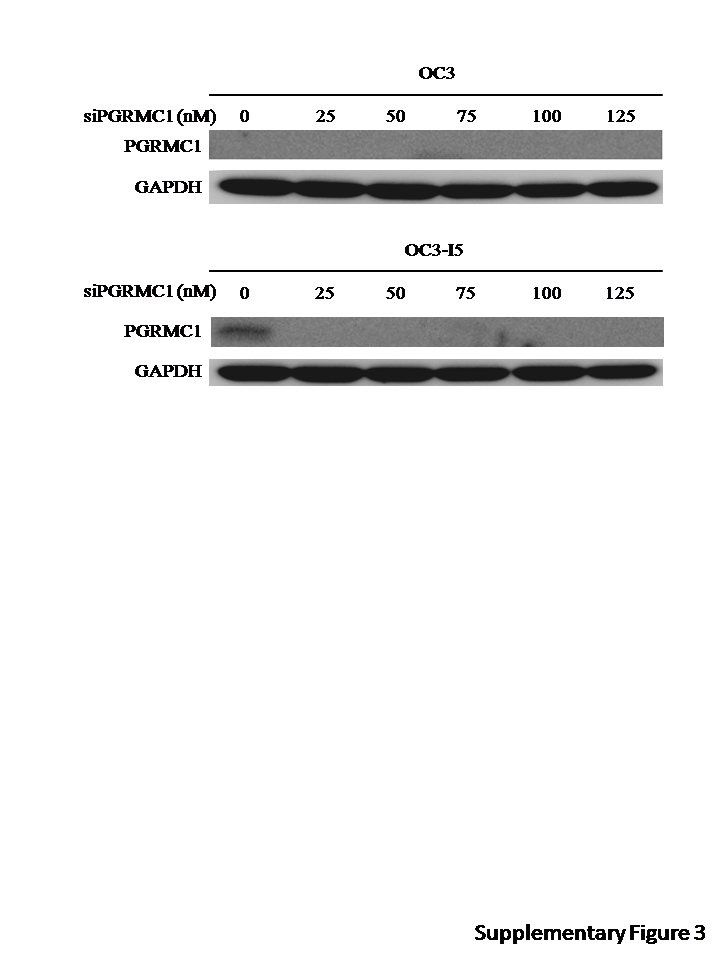

Supplement: Supplementary file 5 — Fig S3 [file JCMM-24-9737-s005.tif]
